# Precise Molecular Engineering of Type I Photosensitizers with Near‐Infrared Aggregation‐Induced Emission for Image‐Guided Photodynamic Killing of Multidrug‐Resistant Bacteria

**DOI:** 10.1002/advs.202104079

**Published:** 2021-12-19

**Authors:** Peihong Xiao, Zipeng Shen, Deliang Wang, Yinzhen Pan, Ying Li, Junyi Gong, Lei Wang, Dong Wang, Ben Zhong Tang

**Affiliations:** ^1^ Center for AIE Research Shenzhen Key Laboratory of Polymer Science and Technology Guangdong Research Center for Interfacial Engineering of Functional Materials College of Materials Science and Engineering Shenzhen University Shenzhen 518060 China; ^2^ Key Laboratory of Optoelectronic Devices and Systems of Ministry of Education and Guangdong Province College of Physics and Optoelectronic Engineering Shenzhen University Shenzhen 518060 China; ^3^ Department of Chemistry Hong Kong Branch of Chinese National Engineering Research Center for Tissue Restoration and Reconstruction The Hong Kong University of Science and Technology Clear Water Bay Kowloon Hong Kong 999077 China; ^4^ Shenzhen Institute of Molecular Aggregate Science and Engineering School of Science and Engineering The Chinese University of Hong Kong, Shenzhen 2001 Longxiang Boulevard, Longgang District Shenzhen City Guangdong 518172 China

**Keywords:** aggregation‐induced emission, multidrug resistance bacteria, photodynamic therapy, photosensitizers, type I reactive oxygen species

## Abstract

Multidrug resistance (MDR) bacteria pose a serious threat to human health. The development of alternative treatment modalities and therapeutic agents for treating MDR bacteria‐caused infections remains a global challenge. Herein, a series of near‐infrared (NIR) anion–*π* ^+^ photosensitizers featuring aggregation‐induced emission (AIE‐PSs) are rationally designed and successfully developed for broad‐spectrum MDR bacteria eradication. Due to the strong intramolecular charge transfer (ICT) and enhanced highly efficient intersystem crossing (ISC), these electron‐rich anion–*π* ^+^ AIE‐PSs show boosted type I reactive oxygen species (ROS) generation capability involving hydroxyl radicals and superoxide anion radicals, and up to 99% photodynamic killing efficacy is achieved for both Methicillin‐resistant *Staphylococcus aureus* (MRSA) and multidrug resistant *Escherichia coli* (MDR *E. coli*) under a low dose white light irradiation (16 mW cm^−2^). In vivo experiments confirm that one of these AIE‐PSs exhibit excellent therapeutic performance in curing MRSA or MDR *E. coli*‐infected wounds with negligible side‐effects. The study would thus provide useful guidance for the rational design of high‐performance type I AIE‐PSs to overcome antibiotic resistance.

## Graphical abstract

A series of near‐infrared anion–*π* ^+^ photosensitizers featuring aggregation‐induced emission (AIE‐PSs) are developed for broad‐spectrum multidrug resistance (MDR) bacteria eradication. These anion–*π* ^+^ AIE‐PSs show boosted type I reactive oxygen species generation capability, achieving a high photodynamic killing efficacy toward both Methicillin‐resistant *Staphylococcus aureus* and MDR *Escherichia coli* under a low dose white light irradiation in vitro and in vivo.

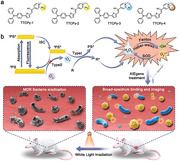

## 1 Introduction

Pathogenic bacterial infections cause severe diseases and rising mortality, which poses a serious threat to global public health.^[^ ^1^ ^]^ Since the discovery of penicillin for the treatment of bacterial infections in 1928, antibiotics have brought dawn for humans to combat bacteria.^[^ ^2^, ^3^ ^]^ However, due to the widespread clinical abuse of antibiotics in the past decades, the emergence of multidrug resistant (MDR) bacteria may bring us back to the “prehistoric era” if we do not have effective antibiotics.^[^ ^4^, ^5^, ^6^ ^]^ Given the circumstance, continuous exploration of new antibiotics is highly desirable, however, involves time‐consuming and costly manipulations. Worse still, the development of new antibiotics usually cannot keep up with the evolution of MDR bacteria, leading to increasingly serious antibiotic resistance.^[^ ^7^, ^8^ ^]^ According to the prediction of the Interagency Coordination Group on Antimicrobial Resistance (IACG), there are at least 700 000 deaths caused by resistant infections every year in the world. If no action is taken, the number will increase to annual 10 million by 2050.^[^ ^9^ ^]^ Evidently, exploiting alternative treatment modalities and therapeutic agents against MDR bacteria are urgently needed.

Photodynamic therapy (PDT), with advantages combination of noninvasiveness, specific spatiotemporal selectivity, and limited drug resistance, has been recognized as an effective and alternative strategy for infection treatment.^[^ ^10^, ^11^, ^12^, ^13^ ^]^ The performance of PDT heavily relies on photosensitizers (PSs) that generate reactive oxygen species (ROS) to inactivate bacteria upon light irradiation.^[^ ^14^, ^15^, ^16^, ^17^ ^]^ Generally, ROS can be divided into two categories: 1) Type I ones including •OH, O_2_^−•^, and H_2_O_2_ produced by electron transfer procedure, and 2) type II one that is ^1^O_2_ generated by energy exchange process.^[^ ^18^, ^19^ ^]^ Compared to the noticeable successes of PDT in the clinical treatment toward cancer, the full promise of antimicrobial PDT, however, has not yet been achieved due to the lack of ideal PSs sharing excellent targeting ability and low side‐effects.^[^ ^20^, ^21^, ^22^, ^23^, ^24^ ^]^ In addition, most traditional fluorescent PSs suffer from aggregation‐caused quenching (ACQ), leading to undesired weak fluorescence intensity and poor ROS generation efficiency, which severely hampers the application of antimicrobial PDT in clinical practice.^[^ ^25^ ^]^ Fortunately, luminogens with aggregation‐induced emission (AIEgens) offer a brand‐new solution, which is a concept established by our research group in 2001.^[^ ^26^, ^27^, ^28^ ^]^ Completely different from the ACQ phenomenon, AIEgens show weak or no emission when dissolved in solutions but remarkably amplified emission in the aggregation state due to the restriction of intramolecular motion (RIM). Although some AIEgen‐mediated antimicrobial PDT protocols have been developed in recent years, the following problems retain to be solved: 1) short absorption and emission wavelengths, which exhibit low tissue penetration and severe photodamage to biosystem; 2) poor imaging ability for Gram negative (G^−^) bacteria, which comes from the insufficient restriction of intramolecular motion of AIEgens by the outer phospholipid membrane of the G^−^ bacteria; 3) low eradication efficiency of drug‐resistant bacteria, especially for MDR negative bacteria, which mainly due to the short lifetime and limited radius of ROS.^[^ ^29^, ^30^, ^31^, ^32^, ^33^, ^34^, ^35^, ^36^, ^37^, ^38^ ^]^

In our previous work, a near infrared (NIR) AIEgen TTPy, which could selectively image and eradicate of Gram‐positive (G^+^) bacteria *Staphylococcus aureus*, was developed.^[^ ^39^ ^]^ This outcome was mainly resulted from the fact that the outer peptidoglycan layer of the G^+^ bacteria can rapidly combine with the positively charged AIE molecules through electrostatic interaction, leading to the restriction of intramolecular motion and fluorescence turn‐on, while the outer phospholipid membrane of the G^−^ bacteria did not have sufficient limitation on the molecular movement of AIEgens.^[^ ^40^ ^]^ Inspired by the application of nitrile groups in drug design to enhance binding affinity with target proteins through hydrogen bond interactions, covalent interactions, polar interactions, *π*–*π* interactions and electrostatic interactions,^[^ ^41^, ^42^ ^]^ we speculate that the introduction of a cyano group into TTPy could further restrict intramolecular motion by forming hydrogen bonds with the outer phospholipid membrane, thereby achieving lighting‐up of both G^−^ and G^+^ bacteria. Besides, the enhanced donor–acceptor (D–A) strength could enormously promote intramolecular charge transfer (ICT), which could facilitate the spatial separation of the highest occupied molecular orbital (HOMO) and the lowest unoccupied molecular orbital (LUMO), resulting in acceleration of intersystem crossing (ISC) process by minimizing energy level difference (Δ*E*_st_) between the singlet state (S_1_) and triplet state (T_1_). Furthermore, anion–*π* ^+^ AIEgens possess an inherent advantage in providing electrons to excited PSs due to the electron‐rich anionic reductant, which is rather critical in the design of free radical‐type PSs.^[^ ^43^, ^44^ ^]^ Therefore, nitrile‐containing anion–*π* ^+^ AIEgens fabricated with strong ICT characteristic not only will achieve broad spectrum imaging of bacteria, but also can eradicate MDR bacteria by generating more destructive type I ROS.

**Scheme 1:**
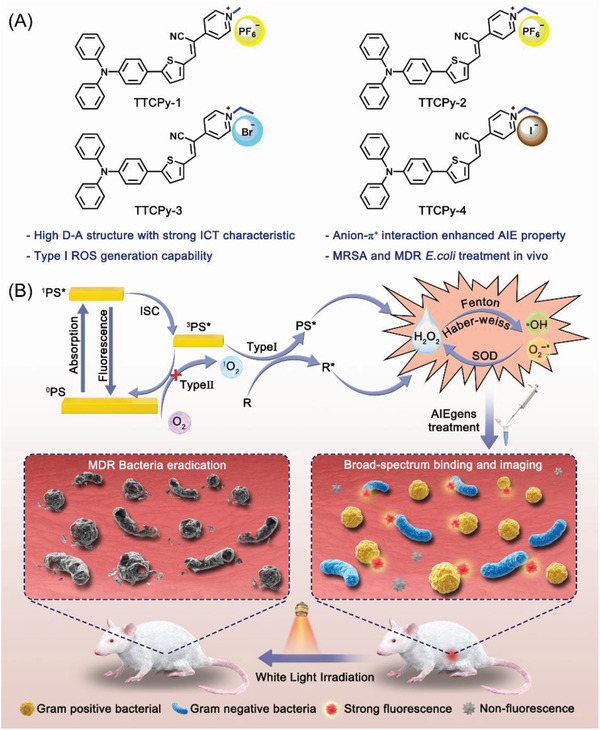
A) Molecular structures of designed type I AIE‐PSs. B) Photophysical and photochemical mechanisms of type‐I and type‐II processes and image‐guided broad spectrum antibacterial application of type I AIE‐PSs.

To verify these hypotheses, we rationally designed four anion–*π* ^+^ AIEgens (TTCPy‐1, TTCPy‐2, TTCPy‐3, and TTCPy‐4) comprising triphenylamine (TPA) fragment (working as an AIE‐active electron donor, D), a thiophene unit (serving as D and *π* bridge), a carbon–carbon double bond (*π* bridge), and cyano and pyridinium moiety (acting as double electron acceptors, A). The D–A strength and ISC process can be precisely regulated by changing the substituents on the pyridine salt and selecting different anions as counterions. The introduction of heavy halide ions in TTCPy‐3 (Br^−^) and TTCPy‐4 (I^−^) can facilitate the effective ISC process due to the efficient enhancement of spin–orbit coupling (SOC) caused by heavy atom effect.^[^ ^45^, ^46^ ^]^ TTCPy‐1, TTCPy‐2, TTCPy‐3, and TTCPy‐4 could achieve not only G^+^ bacteria imaging but also G^−^ bacteria imaging, implying that nitrile‐containing AIEgens indeed enhance the binding ability to phospholipid membrane of G^−^ bacteria. Thanks to the strong ICT intensity and electron‐rich condition contributed by anions, TTCPy series all exhibit highly efficient type I ROS generation capability, offering excellent in vitro/vivo fluorescence imaging guided PDT killing efficiency toward MDR bacteria. We expect this presented design strategy can be applied to the development of more AIE‐active type I PSs to combat bacterial infection (**Scheme** **1**).

## 2 Results and Discussion

### 2.1 Synthesis and Photophysical Properties

Compounds TTCPy‐1, TTCPy‐2, TTCPy‐3, and TTCPy‐4 were synthesized through a few reaction steps. The synthetic routes of these four compounds are depicted in Scheme S1 in the Supporting Information. The common intermediate TTCPy was facile obtained by two steps of Suzuki coupling and condensation reaction using 4‐bromo‐*N*,*N*‐diphenylaniline and (5‐formylthiophen‐2‐yl) boronic acid as starting materials. The alkylation of pyridine in TTCPy using iodomethane and subsequent ion exchange in saturated KPF_6_ solution were successively conducted and achieved a moderate yield of 54% for TTCPy‐1. TTCPy‐2, TTCPy‐3, and TTCPy‐4 were obtained from the corresponding iodoethane and bromoethane in the same procedure with high yields ranging from 58% to 79% (Figures S1–S15, Supporting Information).

**Figure 1:**
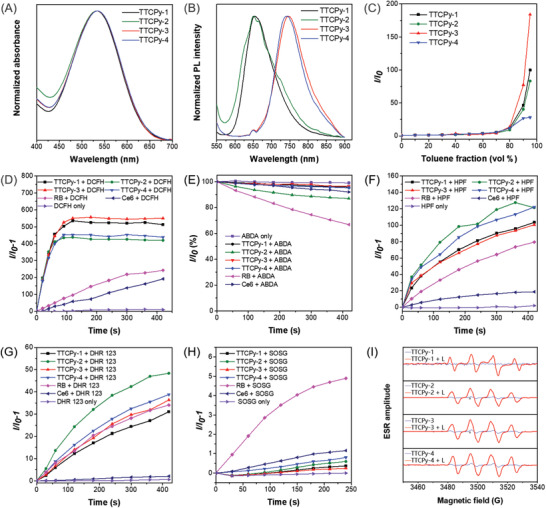
Photophysical properties and measurement of ROS types. A) Normalized absorption spectra of TTCPy‐1, TTCPy‐2, TTCPy‐3, and TTCPy‐4 in the DMSO solution. B) Normalized PL spectra of TTCPy‐1, TTCPy‐2, TTCPy‐3, and TTCPy‐4 in the solid state. C) The plot of the relative emission intensity (*I*/*I*_0_) versus toluene fraction. *I*_0_ and *I* are the peak values of photoluminescence intensities of AIEgens (10 × 10^−6^ m) in DMSO and DMSO/toluene mixtures, respectively. ROS generation of TTCPy‐1, TTCPy‐2, TTCPy‐3, and TTCPy‐4 upon white light irradiation: Relative changes in PL intensity of D) DCFH for overall ROS detection, E) decomposition rates of ABDA for ^1^O_2_ detection, F) HPF for •OH detection, G) DHR 123 for O_2_^−•^ detection, and H) SOSG for ^1^O_2_ detection in the presence of TTCPy‐1, TTCPy‐2, TTCPy‐3, or TTCPy‐4 (1 × 10^−6^ m) upon white light irradiation (16 mW cm^−2^) for different times. I) ESR signals of DMPO for type‐I ROS •OH characterization in the presence of TTCPy‐1, TTCPy‐2, TTCPy‐3, or TTCPy‐4 (0.5 × 10^−3^ m in H_2_O) before and after white light irradiation (100 mW cm^−2^).

With TTCPy series compounds in hands, their optical properties were further evaluated. Different anions have little effect on the maximum absorption peaks, as illustrated in **Figure** **1**A. The maximum absorptions of TTCPy‐1, TTCPy‐2, TTCPy‐3, and TTCPy‐4 in DMSO were located at 532, 532, 534, and 534 nm, respectively. TTCPy‐1 and TTCPy‐2 with hexafluorophosphate (PF_6_^−^) had a maximum emission peak at 654 and 650 nm, respectively (Figure 1B), while the maximum emission peak of TTCPy‐3 and TTCPy‐4 with bromide (Br^−^) and iodide (I^−^) as counterions red‐shifted to 748 and 742 nm, respectively, which may be ascribed to the anion–*π* ^+^ interactions between the heavy halide ions and the positively charged pyridine rings.^[^ ^47^, ^48^, ^49^ ^]^ Photoluminescence (PL) spectra of the TTCPy series were then measured in DMSO/toluene mixtures to evaluate their AIE tendency. As depicted in Figure 1C and Figure S16 in the Supporting Information, when the toluene fraction (*f*_T_) was below 70%, the PL intensities of these four compounds were quite weak, with negligible fluorescence quantum yields ranging from 0.5% to 0.9% in solution. Subsequently, increasing *f*_T_ to 80%, the PL intensities of TTCPy‐1, TTCPy‐2, TTCPy‐3, and TTCPy‐4 amplified dramatically and reached to their maximum values at 99% of *f*_T_, indicating their typical AIE features. Due to the twist intramolecular charge transfer (TICT) effect of TTCPy series, a slight blue shift in fluorescence emission was observed with the decrease in polarity of the solvent mixtures. It is noteworthy that TTCPy‐3 with bromide (Br^−^) had the highest *α*_AIE_ value up to 184, which may be attributed to much stronger anion–*π* ^+^ interactions between the bromide anions and the positively charged pyridine ring that avoid *π*–*π* stacking in the aggregation state. As shown in Table S1 in the Supporting Information, the fluorescence quantum yields of TTCPy‐1, TTCPy‐2, TTCPy‐3, and TTCPy‐4 reached 12.9%, 12.1%, 19.2%, and 6.1%, respectively, in the aggregation state, which were about 14.3‐, 15.1‐, 38.4‐, and 8.7‐fold of those in DMSO solution (0.9%, 0.8%, 0.5%, and 0.7%, respectively), further confirming their AIE characteristics.

### 2.2 ROS Generation Evaluation and Theoretical Explanation

The development of high‐performance photosensitizers highly depends on the ISC rate and its ability in light absorption.^[^ ^50^ ^]^ On one hand, ISC can be significantly amplified through HOMO–LUMO engineering to achieve a small energy gap between S_1_ and T_1_. On the other hand, ISC can be increased by incorporation of heavy atoms into their structures, which can strengthen the spin‐orbit coupling to boost the triplet quantum yields. It is generally accepted that electron‐rich anion–*π* ^+^ AIEgens with strong ICT effect can generate more free radical ROS via type I mechanism.^[^ ^43^, ^44^ ^]^ In addition, the suppressed molecular motions of AIEgens in the aggregation state are beneficial for ISC process, which is called as the aggregation‐induced ISC.^[^ ^51^ ^]^ Therefore, as an elegant combination of all the above mentioned factors, TTCPy series are expected to be high performance AIE‐active type I photosensitizers.

The total ROS‐generating abilities of TTCPy‐1, TTCPy‐2, TTCPy‐3, and TTCPy‐4 in DMSO solution with 99% phosphate‐buffered saline (PBS) fractions were investigated using 2′,7′‐dichlorodihydrofluo‐rescein (DCFH) as an indicator under white light irradiation. As shown in Figure 1D and Figure S17 in the Supporting Information, the fluorescence intensity of DCFH rapidly enhanced in the presence of these AIE‐PSs along with the continuous irradiation due to the greatly suppressed nonradiative decay in the aggregation state, while negligible increase in fluorescence signal was detected for the irradiated solution with DCFH alone. Thanks to the strong heavy atom effect of bromine and iodine ions which can promote the ISC process, TTCPy‐3 and TTCPy‐4 had higher ROS generation ability than TTCPy‐2 with an emission intensity nearly 550‐fold higher than the initial intensity after 2 min white light irradiation. In addition, all these AIE‐PSs showed much better overall ROS generation capability compared with commercially available Rose Bengal (RB) and Chlorine 6 (Ce6), the latter two are both very popular and reputable PSs.

We systematically investigated the species of ROS to have an in‐depth understanding of the mechanism of ROS generated by these AIE‐PSs. Initially, 9,10‐anthracenediyl‐bis(methylene) dimalonic acid (ABDA), a common used indicator was utilized for the detection of ^1^O_2_ generation by these AIE‐PSs. The absorption signal of ABDA treated with TTCPy‐1, TTCPy‐2, TTCPy‐3, and TTCPy‐4, respectively, decreased about 4.19%, 13.11%, 3.36%, and 4.78%, which was obviously lower than that treated with the commercial RB (33.22%), implying their insufficient ^1^O_2_ generation capacity (Figure 1E; Figure S18, Supporting Information). The •OH generation efficiency of these AIE‐PSs was determined by comparing with RB and Ce6 under the same circumstances by using hydroxyphenyl fluorescein (HPF) as indicator. As illustrated in Figure 1F and Figure S19 in the Supporting Information, the fluorescence intensity of HPF enhanced more than 80‐fold under continuous irradiation for 7 min in the presence of these AIE‐PSs, further demonstrating that all of these AIE‐PSs were capable of generating •OH effectively through type‐I mechanism, while there was almost no emission for HPF alone under white light irradiation. Furthermore, a superoxide anion radical (O_2_^−•^) probe, dihydrorhodamine 123 (DHR123), was used to estimate the O_2_^−•^ generation ability of these AIE‐PSs. As displayed in Figure 1G and Figure S20 in the Supporting Information, the PL intensity of DHR 123 in the TTCPy‐2 and TTCPy‐4 group after 7 min white light irradiation was over 48‐fold and 38‐fold of that before the irradiation, respectively, suggesting significantly high O_2_^−•^ generation efficiency of TTCPy‐2 and TTCPy‐4. Noteworthily, the O_2_^−•^ generation capability of TTCPy‐1 (31‐fold) and TTCPy‐3 (36‐fold) was comparable with RB (34‐fold), while significantly superior to Ce6 (2‐fold). In addition, singlet oxygen sensor green (SOSG) was also utilized to detect type II ROS ^1^O_2_. There was almost no change of SOSG fluorescence in the presence of these AIE‐PSs upon white light irradiation (Figure 1H), reflecting their infirm ^1^O_2_ production efficiencies. The results from SOSG and ABDA indicators were consistent, which strongly confirmed the weak singlet oxygen generation ability and high free radical production capability from type I mechanism of these AIE‐PSs.

To further confirm the •OH and O_2_^−•^ production, 5,5‐dimethyl‐1‐pyrroline‐*N*‐oxide (DMPO) was used as the spin‐trap agent to perform electron spin resonance (ESR) measurement. In the presences of DMPO and AIE‐PSs, the resultant ESR spectrum displayed a typical four‐line resonances with 1:2:2:1 intensity under white light irradiation (Figure 1I), which is the characteristic resonances for DMPO/•OH adduct. In addition, obvious six‐line ESR signal was also identified, which origin from DMPO/O_2_^−•^ adduct (Figure S21, Supporting Information).

To profoundly study the mechanism of type I ROS generation, time‐dependent density functional theory (TD‐DFT) was carried out based on the B3LYP‐D3/6‐31G (d,p) level. As shown in Figure S22 in the Supporting Information, the electron clouds of HOMOs of these AIE‐PSs were mainly delocalized at the TPA moiety, while the LUMOs were primarily contributed by the orbitals of the cyano and pyridinium groups, suggesting the separation of HOMOs–LUMOs and their typical D–A characteristics. HOMOs–LUMOs energy gaps of TTCPy‐1, TTCPy‐2, TTCPy‐3, and TTCPy‐4 were determined to be 2.17, 2.14, 2.17, and 1.81 eV, respectively. The Δ*E*_st_ of TTCPy‐4 (0.74 eV) with iodide (I^−^) as counterions was the smallest among these AIE‐PSs (1.22, 1.20, and 1.15 eV for TTCPy‐1, TTCPy‐2, and TTCPy‐3, respectively), implying that TTCPy‐4 could generate free radicals more efficiently, which is in good accordance with the experimental results (Figure 1E). We further investigated electrochemical properties of TTCPy series by cyclic voltammetry using ferrocene (Fc) as the external standard to help us understand why these AIE‐PSs are prone to generating O_2_^−•^ and •OH via the type I process. As depicted in Figure S23 in the Supporting Information, the oxidation potentials of TTCPy‐1, TTCPy‐2, TTCPy‐3, and TTCPy‐4 were located at 0.767, 0.709, 0.633, and 0.234 eV, respectively, demonstrating the stronger electron‐donating character of both I^−^ and Br^−^ ions, which endowed the excited TTCPy‐3 and TTCPy‐4 PSs with easier access to electrons from electron‐rich environment than TTCPy‐1 and TTCPy‐2, and then transfer an electron to molecular oxygen through type I mechanism. Therefore, the above theoretical and experimental results support our design concept that type I ROS can be generated by electron‐rich anion–*π* ^+^ AIE‐PSs with enhanced ICT intensity and ISC process via acceptor engineering.

### 2.3 Broad Spectrum Bacteria Imaging and Light‐Enhanced Photodynamic Antibacterial Study

**Figure 2:**
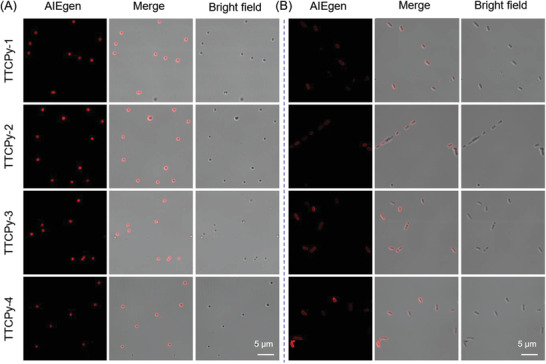
CLSM images of bacteria treated with AIEgens. A) Bright‐field and fluorescent images of MRSA incubated with 10 × 10^−6^ m of TTCPy‐1, TTCPy‐2, TTCPy‐3, and TTCPy‐4 for 5 min. B) Bright‐field and fluorescent images of *E. coli* treated with 10 × 10^−6^ m of TTCPy‐1 (10 min), TTCPy‐2 (10 min), TTCPy‐3 (15 min), and TTCPy‐4 (15 min) for different times. Ex = 488 nm, Em = 600–700 nm.

Since the nitrile‐containing TTCPy series could enhance the binding affinity to the phospholipid membrane of bacterial cell walls through hydrogen bond interactions,^[^ ^42^ ^]^ leading to the restriction of the intramolecular motions of AIEgens and rapid enhancement of emission intensity, they are expected to realize the broad‐spectrum imaging of G^−^ and G^+^ bacteria. We selected *S. aureus* and *Escherichia coli* as representatives of G^+^ and G^−^ bacteria respectively. Confocal laser scanning microscopy (CLSM) showed that upon incubating *S. aureus* with 10 × 10^−6^ m AIE‐PSs, the bright red fluorescence was clearly observed within 5 min with excellent contrast to the background (**Figure** **2**A), indicating that these positive charged AIE‐PSs have high binding affinity to peptidoglycan on the cell membrane of G^+^ bacteria. Different from G^+^ bacterial, the outer membrane in G^−^ bacteria is a layer of the phospholipid membrane, which is an effective barrier to prevent the direct contact of extraneous invaders with the interbedded peptidoglycan network. Therefore, when incubated with previously reported AIEgens, there should be only weak fluorescence emission at the beginning.^[^ ^40^ ^]^ After 10 min incubation of *E. coli* with TTCPy‐1, TTCPy‐2, TTCPy‐3, and TTCPy‐4, the fluorescent signals were clearly visualized in the bacteria. *E. coli* stained with TTCPy‐3 and TTCPy‐4 exhibited bright fluorescence with the prolongation of incubation time to 15 min. These results revealed that TTCPy series could achieve the broad‐spectrum imaging of bacteria (Figure 2B).

**Figure 3:**
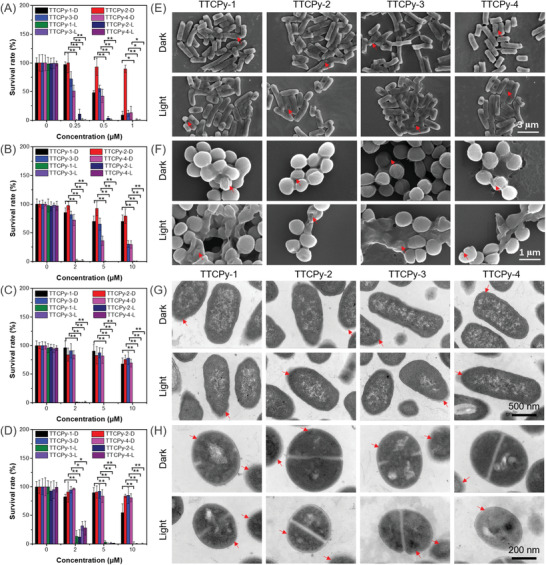
Antibacterial activity and visualizing TTCPy‐induced morphological changes of bacteria with or without white light irradiation. Survival rate of A) MRSA, B) *E. coli*, C) *E. coli* Top 10, and D) MDR *E. coli* cultured on agar plate supplemented TTCPy‐1, TTCPy‐2, TTCPy‐3, and TTCPy‐4 at different concentrations in darkness (D) or upon white light irradiation (L) (16 mW cm^−2^) for 10 min (*n* = 3). The data show significant statistical differences between MRSA, *E. coli*, *E. coli* Top 10, and MDR *E. coli*‐treated by TTCPy‐1, TTCPy‐2, TTCPy‐3, and TTCPy‐4 with or without light irradiation (16 mW cm^−2^), respectively (^*^ *P* < 0.05 and ^**^ *P* < 0.001). Visualizing TTCPy‐1, TTCPy‐2, TTCPy‐3, and TTCPy‐4‐induced morphological changes of E) *E. coli* and F) MRSA in darkness or upon white light irradiation (16 mW cm^−2^) by FE‐SEM. TEM images of TTCPy‐1, TTCPy‐2, TTCPy‐3, and TTCPy‐4‐induced morphological changes of G) *E. coli* and H) MRSA in darkness or upon white light irradiation (16 mW cm^−2^). The red arrows represent bacteria with collapsed and destroyed membrane.

Encouraged by the broad‐spectrum imaging capability and high type I ROS (•OH and O_2_^−•^) generation efficiency of these AIE‐PSs, their antibacterial activity against MRSA, *E. coli*, *E. coli* Top 10, and MDR *E. coli* were investigated through the plate count method (**Figure** **3**A–D; Figures S24–S27, Supporting Information). In the absence of AIE‐PSs, there was no obvious change in the survival rates of MRSA, *E. coli*, *E. coli* Top 10, and MDR *E. coli* in the dark or under white light irradiation. About 90% of MRSA incubated with TTCPy‐1, TTCPy‐3, and TTCPy‐4 were killed at a concentration of 1 × 10^−6^ m under dark condition, while TTCPy‐2 had a low MRSA killing efficacy under the same conditions (Figure 3A). Compared with TTCPy‐1 and TTCPy‐2, both TTCPy‐3 and TTCPy‐4 had higher killing efficacies toward *E. coli* with a survival rate of 30% in the dark (Figure 3B). The dark toxicity of these AIE‐PSs to bacteria is the direct evidence of the interaction between nitrile‐containing AIEgens and the bacterial cell walls. For drug‐resistant bacterial *E. coli* Top 10 and MDR *E. coli*, the dark toxicity of these AIE‐PSs is significantly reduced, with the survival rate of bacteria as high as 80% even at a concentration of 10 × 10^−6^ m (Figure 3C,D). Upon white light irradiation, more than 99% of MRSA were killed at a low concentration of 0.25 × 10^−6^ m of TTCPy series except for TTCPy‐2, suggesting a high photodynamic killing efficiency of TTCPy‐1, TTCPy‐3, and TTCPy‐4 toward MRSA. This outcome can be reasonably attributed to the more efficient type I ROS generations of TTCPy‐1, TTCPy‐3, or TTCPy‐4 than that of TTCPy‐2 (Figure 1E). The survival rates of *E. coli* and *E. coli* Top 10 incubated with 2 × 10^−6^ m TTCPy‐3 descended to less than 1% under white light irradiation (16 mW cm^−2^). When the concentration of AIE‐PSs was increased to 5 × 10^−6^ m, almost 100% of the bacteria were eradicated including the tricky MDR *E. coli*. For the control group without these AIE‐PSs, there were almost no changes in the morphology of both *E. coli* and MRSA under white light irradiation (16 mW cm^−2^) visualized by field emission scanning electron microscopy (FE‐SEM) and transmission electron microscope (TEM) (Figure S28, Supporting Information). The results indicate that these AIE‐PSs have a profound and broad‐spectrum PDT killing efficacy on MDR bacteria.

Cytotoxicity is a key parameter in the development of antibacterial PSs. An ideal antibacterial PS should not cause any damage to mammalian cells at the working concentrations. The biocompatibility of these AIE‐PSs toward human umbilical vein endothelial cells (HUVECs) and healthy NIH‐3T3 cells were evaluated by MTT assay. As shown in Figure S29 in the Supporting Information, there was negligible toxicity toward healthy HUVECs with the increasing concentration of AIE‐PSs (0.315 × 10^−6^, 0.625 × 10^−6^, 1.25 × 10^−6^, 2.5 × 10^−6^ , and 5 × 10^−6^ m) both in the dark and under the light irradiation, while the cell viability decreased obviously when treated with 10 × 10^−6^ m TTCPy‐1 and TTCPy‐4 under light irradiation. The same trend of cell viability were observed in healthy NIH‐3T3 cells incubated with these four AIE‐PSs, indicating that TTCPy‐2 and TTCPy‐3 had a better performance in biocompatibility (Figure S30, Supporting Information).

### 2.4 The Mechanism of the Bacteria Inhibition

To investigate the antibacterial mechanism and gain more insights into the structure–function relationships of TTCPy series, we utilized FE‐SEM and TEM to visualize the morphological changes of the bacteria incubated with TTCPy‐1, TTCPy‐2, TTCPy‐3, and TTCPy‐4 in dark or under white light irradiation, selecting *E. coli* (G^−^) and MRSA (G^+^) as representative bacterials. *E. coli* in the AIE‐PSs@Light groups showed obvious caves on the cell walls, while the morphology of bacteria in the dark barely changed (Figure 3E). As displayed in Figure 3F, the cell walls of MRSA collapsed dramatically in the presence of AIE‐PSs with white light irradiation (16 mW cm^−2^). Additionally, TEM analysis was performed to visualize the morphological features of *E. coli* and MRSA before and after PDT treatment. It was observed that the edge of *E. coli* cell wall became blurred under AIE‐PSs and light irradiation compared with the control groups (Figure 3G). The outer surfaces of MRSA bacteria incubated with the AIE‐PSs were destroyed more obviously in the presence of light irradiation (Figure 3H). The antibacterial effects of these AIE‐PSs are mainly originated from the inactivation of biomacromolecules by free radicals generated from type I mechanism upon white light irradiation.

### 2.5 In Vivo Anti‐Infection Assay

**Figure 4:**
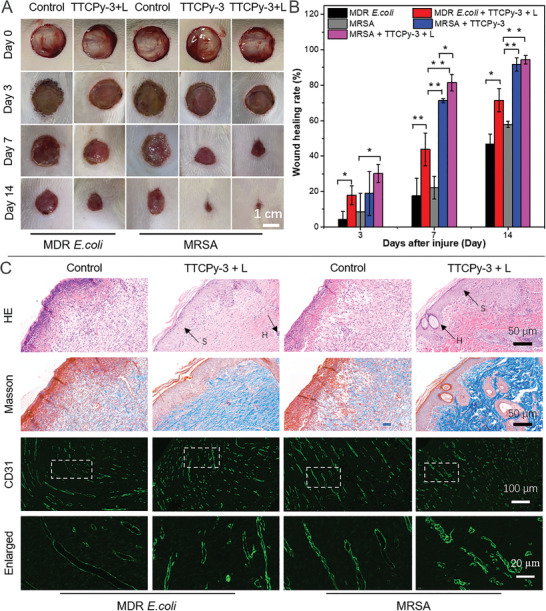
In vivo evaluation of TTCPy‐3 in treatment of MDR *E. coli*‐ and MRSA‐infected wounds on rats. A) Photographs of infected wounds treated by TTCPy‐3 or TTCPy‐3 plus white‐light irradiation after injury for different time periods. B) The proportion of the MDR *E. coli*‐ and MRSA‐infected wound area on day 3, day 7, and day 14 after the injury (*n* = 3). The data show significant statistical differences between MDR *E. coli*‐ and MRSA‐infected groups and MDR *E. coli*‐ and MRSA‐infected wounds treated by TTCPy‐3 with or without light irradiation, and MRSA‐infected wounds treated by TTCPy‐3 with or without light irradiation (16 mW cm^−2^) (^*^ *P* < 0.05 and ^**^ *P* < 0.001). C) HE, Masson, and CD 31 staining of the sectioned tissues of wounds on rats. MDR *E. coli*‐ and MRSA‐infected wounds were treated with or without TTCPy‐3 plus white‐light irradiation. The tissues were resected on day 14 after injury. The letters in the images indicate specific cell types and structures in the histological sections. H, hair follicle; S, squamous epithelial cell.

Based on the in vitro antibacterial results of these AIE‐PSs, TTCPy‐3 was selected to further evaluate the antibacterial effect in vivo. Animal models with MRSA and MDR *E. coli*‐infected wounds on the dorsal skin of Wistar rats were established, respectively. For the rats infected with MDR *E. coli*, the rats were randomly divided into two groups: 1) treated with PBS only (control group); 2) TTCPy‐3@white‐light (PDT group). Considering the excellent dark toxicity of TTCPy‐3 to G^+^ bacteria, the rats infected with MRSA were randomly assigned to three groups for the following treatments: PBS only (control group), TTCPy‐3 only (dark group), and TTCPy‐3@white‐light (PDT group) (**Figure** **4**A).

In order to clearly observe the healing process, the macroscopic appearance of the wounds were captured at different time points. At day 3 postinfection, there was no significant difference in all the wounds. At day 7, the sizes of the MDR *E. coli*‐infected wounds treated with TTCPy‐3@white‐light irradiation (16 mW cm^−2^, 30 min) was apparently smaller than that of the control group, while the sizes of MRSA‐infected wounds treated with TTCPy‐3 only (dark group) and TTCPy‐3@white‐light irradiation (16 mW cm^−2^, 30 min, PDT group) were significantly smaller in comparison to the control group treated with PBS only. At day 14, the MDR *E. coli*‐infected wounds treated with TTCPy‐3@white‐light irradiation were much smaller and cleaner than the control group, and the wound healing rate was over 70%. Meanwhile, the MRSA‐infected wounds treated with TTCPy‐3 only and TTCPy‐3@white light had almost recovered with a wound healing rate up to 91.7% and 94.4%, respectively (Figure 4B). The MRSA‐infected wounds treated with TTCPy‐3 only had a good wound‐healing effect mainly due to the dark toxicity, which is in good accordance with the in vitro killing efficiency of TTCPy‐3 toward MRSA under dark condition (Figure 3A). Moreover, hematoxylin and eosin (H&E) and Masson staining of the sectioned tissues of the wounds on rats were utilized to evaluate the wound‐healing efficacy.^[^ ^52^ ^]^ As clearly shown in Figure 4C, at day 14, intact and thick epidermis appeared in the neonatal skin of the PDT groups. Moreover, hair follicles and squamous epithelial cells can be clearly observed on the sectioned tissues of the wounds from the same group, strongly indicating the excellent wound healing efficacy of TTCPy‐3 in the presence of light irradiation. Similarly, the collagen fibers, which were dyed blue, could be visualized clearly in the neonatal skin after Masson's trichrome staining, demonstrating a much more prominent antibacterial efficiency in the PDT groups. Finally, CD31 staining was performed and the images indicated that more blood vessels were observed in the neonatal structure of PDT groups. Therefore, the in vivo results strongly demonstrated that TTCPy‐3 has a supremely prominent light‐enhanced antimicrobial effect toward MDR bacteria and can dramatically promote the wound‐healing process, which is highly consistent with antibacterial tests in vitro.

## 3 Conclusions

In summary, we developed a series of NIR anion–*π* ^+^ AIE‐PSs (TTCPy‐1, TTCPy‐2, TTCPy‐3, and TTCPy‐4) with efficient ROS generation via the type I mechanism, which can be used at very low concentrations to effectively kill drug‐resistant bacteria strains. Theoretical calculations and experimental results suggested that strong D–A effect and sufficient small Δ*E*_ST_ play dominate roles in the ISC process, which is in favor of producing destructive type I ROS. Thanks to the broad‐spectrum bacterial binding ability and efficient type I ROS generation capability, TTCPy series exhibited excellent photodynamic antibacterial activity in destroying MRSA, *E. coli*, *E. coli* Top 10, and MDR *E. coli* at low concentration (0.25 × 10^−6^ m for MRSA, 2 × 10^−6^ m for *E. coli* and *E. coli* Top 10, and 5 × 10^−6^ m for MDR *E. coli*) under a low white light dose (16 mW cm^−2^). Benefiting from its excellent biocompatibility and bright emission in the NIR region, TTCPy‐3 has been utilized for photodynamic inactivation of MRSA and MDR *E. coli* in vivo, leading to successfully suppression of the bacterial infections in wounds. Hence, this study provides a new strategy for the design of type I AIE photosensitizers as next‐generation advanced antimicrobial agents.

## Conflict of Interest

The authors declare no conflict of interest.

## Supporting information

Supporting Information

## Data Availability

The data that support the findings of this study are available from the corresponding author upon reasonable request.
