# Supplementary material for: Precise Molecular Engineering of Type I Photosensitizers with Near‐Infrared Aggregation‐Induced Emission for Image‐Guided Photodynamic Killing of Multidrug‐Resistant Bacteria
Source: Adv Sci (Weinh). 2021 Dec 19;9(5):2104079. doi: 10.1002/advs.202104079 (PMC8844491; doi:10.1002/advs.202104079)
Supplement: Supplementary file 1 — Supporting Information [file ADVS-9-2104079-s001.pdf]

ADVANCED SCIENCE
Open Access

Supporting Information

for *Adv. Sci.*, DOI: 10.1002/advs.202104079

Precise Molecular Engineering of Type I Photosensitizers with Near-Infrared Aggregation-Induced Emission for Image-Guided Photodynamic Killing of Multidrug-Resistant Bacteria

Peihong Xiao, Zipeng Shen, Deliang Wang, Yinzhen Pan, Ying Li, Junyi Gong, Lei Wang, Dong Wang* and Ben Zhong Tang**

Supporting Information

Precise Molecular Engineering of Type I Photosensitizers with Near-Infrared Aggregation-Induced Emission for Image-Guided Photodynamic Killing of Multidrug-Resistant Bacteria

Peihong Xiao, Zipeng Shen, Deliang Wang, Yinzhen Pan, Ying Li, Junyi Gong, Lei Wang, Dong Wang* and Ben Zhong Tang**

Table of Contents

Experimental Procedures 2

Materials and instruments 2

Synthesis and Characterization 3

Total ROS Detection in Aqueous Solution 7

$^{\bullet}$OH Detection in Aqueous Solution 7

$O_2^{-\bullet}$ Detection in Aqueous Solution 8

$^{1}O_2$ Detection in Aqueous Solution 8

ESR Analysis 9

Theoretical calculations 10

Cyclic Voltammetry Measurement 10

Bacteria Culture 11

Bacteria Staining and Imaging 11

Antimicrobial Assay 11

FE-SEM Analysis 12

TEM Analysis 13

Cell culturing and staining 13

Cell viability via MTT Assay 14

***In Vivo* Anti-Infection Assay** 14

Histological Analysis 15

Statistical Analysis 16

Figures and tables 16

References 36

Experimental Procedures

Materials and instruments

All solvents and chemicals, unless special stated, were purchased commercially in analytical grade and used without further purification. Luria-Bertani (LB) broth and LB agar were from Solarbio (China). Phosphate buffer saline (PBS) was from Sigma-Aldrich. *Escherichia coli* (*E. coli*) (ATCC 12228) were from ATCC. The Ampr Ampicillin *E. coli* (*E. coli* Top 10) was from Beijing Bio-Med Technology Development Co., Ltd. MDR *E. coli* and MRSA were from Beijing Tiantan Hospital (China).^{1}H and ^{13}C spectra were measured on a Bruker ARX 500 (or 600) NMR spectrometer using DMSO-d_6 and $CDCl_3$ as the deuterated solvent, respectively. Tetramethylsilane (TMS; δ = 0 ppm) was used as the internal standard. High-resolution mass spectra (HRMS) were recorded on a Finnegan MAT TSQ 7000 Mass Spectrometer System. UV-vis absorption spectra were taken on a PerkinElmer Lambda 950 spectrophotometer. PL spectra were recorded on an Edinburgh FS5 fluorescence spectrophotometer and Milton Roy Spectronic 3000 array spectrophotometer. ESR analysis was performed on a Bruker EMS^{plus}-10/12 spectrometer. The absolute PLQY was determined by a Hamamatsu quantum yield spectrometer C11347 Quantaurus QY. The cell viability was conducted by the MTT assay. The cellular fluorescence images were taken by a confocal laser scanning microscope (CLSM, ZEISS-LSM900). Intermediate compounds **1** was synthesized according to the literature method.[1]

Synthesis and Characterization

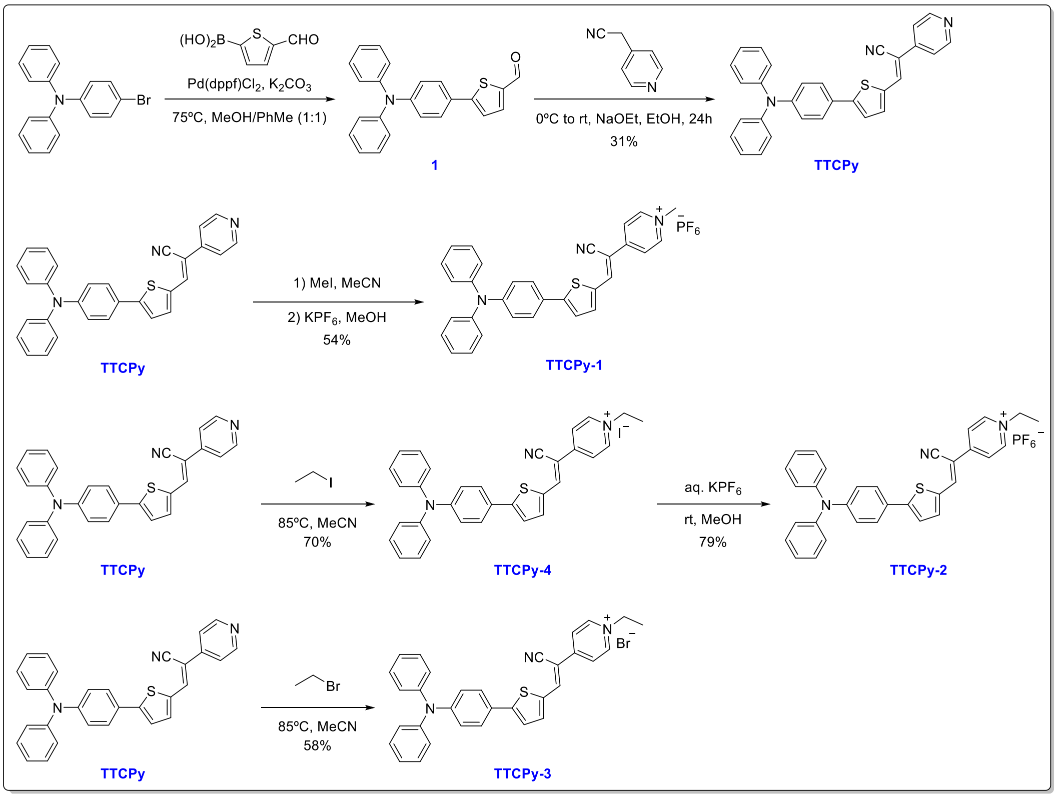

Scheme S1. The synthetic routes of AIEgens.

Synthesis of TTCPy:

To a solution of compound **1** (100 mg, 0.281 mmol) and 4-Pyridylacetonitrile (33 mg, 0.281 mmol) in EtOH, NaOEt (1.9 mg, 0.028 mmol) was added at 0°C. The mixture was stirred at room temperature for 24 h, followed by solvent removal. The residue was purified by silica gel chromatography to generate an orange solid with a yield of 31% (40 mg). ^{1}H NMR (500 MHz, Methylene Chloride-d_2) δ 8.63 (d, J = 6.3 Hz, 2H), 7.85 (s, 1H), 7.65 (d, J = 4.0 Hz, 1H), 7.56 (d, J = 8.7 Hz, 2H), 7.53 – 7.47 (m, 2H), 7.36 – 7.24 (m, 5H), 7.18 – 7.12 (m, 4H), 7.10 (t, J = 7.4 Hz, 2H), 7.08 – 7.00 (m, 2H). ^{13}C NMR (126 MHz, Methylene Chloride-d_2) δ

151.72, 150.88, 149.27, 147.46, 141.97, 137.27, 137.00, 135.76, 129.85, 127.45, 126.62, 125.54, 124.21, 123.25, 122.80, 119.73, 117.80, 104.10. HRMS (MALDI-TOF): m/z: [M+H] + calcd for $C_{30}H_{21}N_3S$: 455.1456, found: 456.1540.

Synthesis of TTCPy-1:

To a solution of TTCPy (40 mg, 0.088 mmol) in dry acetonitrile (3 mL) was added MeI (174 mg, 1.23 mmol), the mixture was refluxed at 85°C overnight. After cooling to room temperature, the mixture was poured into diethyl ether. The dark red precipitates formed were filtered by suction filtration. The precipitates were re-dissolved in MeOH (4 mL) and mixed with saturated KPF_6 solution (3 mL). After stirring for 1 h at room temperature, the methanol was evaporated by compressed air. The red precipitates were filtered again, washed with water and dried under reduced pressure. The residue was purified by a neutral aluminum oxide column using DCM and methanol mixture (50:1 v/v) as eluting solvent to give a dark red powder of TTCPy-1 with a yield of 54% (29 mg). ^{1}H NMR (600 MHz, DMSO-d_6) δ 8.93 (m, 3H), 8.27 (d, J = 7.2 Hz, 2H), 7.97 (d, J = 4.1 Hz, 1H), 7.74 – 7.69 (m, 3H), 7.40 – 7.36 (m, 4H), 7.16 (t, J = 7.4 Hz, 2H), 7.12 (d, J = 7.3 Hz, 4H), 6.98 (d, J = 8.8 Hz, 2H), 4.29 (s, 3H). ^{13}C NMR (151 MHz, DMSO-d_6) δ 154.30, 149.09, 149.00, 146.23, 145.40, 143.66, 141.93, 134.62, 129.84, 127.62, 125.24, 124.96, 124.41, 121.99, 121.36, 116.66, 98.93, 47.03. HRMS (MALDI-TOF): m/z: [M+H] + calcd for $C_{31}H_{24}N_3S^+$: 470.1690, found: 470.1685.

Synthesis of TTCPy-4:

To a solution of TTCPy (100 mg, 0.220 mmol) in dry acetonitrile (10 mL) was added Iodoethane (103 mg, 0.659 mmol), the mixture was refluxed at 85°C overnight. After cooling to room temperature, the mixture was poured into diethyl ether. The dark red precipitates formed were filtered by suction filtration, washed with Et_2O (10 mL x 3) and dried under reduced pressure to give a dark powder of TTCPy-4 with a yield of 70% (94 mg). ^1H NMR (500 MHz, DMSO-d_6) δ 9.05 (d, J = 7.0 Hz, 2H), 8.96 (s, 1H), 8.30 (d, J = 7.0 Hz, 2H), 7.99 (d, J = 4.0 Hz, 1H), 7.72 (dd, J = 6.5, 2.3 Hz, 2H), 7.40 – 7.35 (m, 4H), 7.18-7.14 (m, 2H), 7.13-7.09 (m, 5H), 6.98 (d, J = 8.8 Hz, 2H), 4.59 (q, J = 7.3 Hz, 2H), 1.54 (t, J = 7.3 Hz, 3H). ^{13}C NMR (126 MHz, DMSO-d_6) δ 154.35, 149.41, 149.01, 146.22, 144.36, 143.77, 141.98, 134.63, 129.84, 127.64, 125.23, 124.98, 124.87, 124.41, 122.38, 121.36, 116.65, 98.92, 55.45, 16.17. HRMS (MALDI-TOF): m/z: [M+H] + calcd for $C_{32}H_{26}N_3S^+$: 484.1842, found: 484.1847.

Synthesis of TTCPy-2:

TTCPy-4 (100 mg, 0.164 mmol) was dissolved in MeOH (10 mL) and mixed with saturated KPF_6 solution (10 mL). After stirring for 1 h at room temperature, methanol was evaporated by compressed air. The dark red precipitates were filtered again, washed with water and dried under reduced pressure. The residue was purified by a neutral aluminum oxide column using DCM and methanol mixture (50:1 v/v) as eluting solvent to give a red powder of TTCPy-1

with a yield of 79% (82 mg). ^{1}H NMR (500 MHz, DMSO-d_6) δ 9.04 (d, J = 6.8 Hz, 2H), 8.95 (s, 1H), 8.29 (d, J = 7.1 Hz, 2H), 7.98 (d, J = 4.2 Hz, 1H), 7.77 – 7.69 (m, 3H), 7.40 – 7.36 (m, 4H), 7.16 (t, J = 7.5 Hz, 2H), 7.13 (d, J = 7.2 Hz, 4H), 6.99 (d, J = 8.8 Hz, 2H), 4.57 (q, J = 7.3 Hz, 2H), 1.54 (t, J = 7.3 Hz, 3H). ^{13}C NMR (126 MHz, DMSO-d_6) δ 154.37, 149.42, 149.03, 146.23, 144.37, 143.77, 141.96, 134.62, 129.84, 127.64, 125.24, 124.95, 124.44, 124.42, 122.38, 121.37, 116.66, 98.94, 55.46, 16.17. HRMS (MALDI-TOF): m/z: [M+H] + calcd for $C_{32}H_{26}N_3S^+$: 484.1842, found: 484.1847.

Synthesis of TTCPy-3:

To a solution of TTCPy (100 mg, 0.220 mmol) in dry acetonitrile (10 mL) was added bromoethane (72 mg, 0.659 mmol), the mixture was refluxed at 85°C overnight. After cooling to room temperature, the mixture was poured into diethyl ether. The dark precipitates formed were filtered by suction filtration, the precipitates were further purified by a neutral aluminum oxide column using DCM and methanol mixture (50:1 v/v) as eluting solvent to give a dark powder of TTCPy-3 with a yield of 58% (72 mg). ^{1}H NMR (600 MHz, DMSO-d_6) δ 9.07 (d, J = 6.8 Hz, 2H), 9.03 (s, 1H), 8.31 (d, J = 7.2 Hz, 2H), 8.01 (d, J = 4.1 Hz, 1H), 7.75 – 7.69 (m, 3H), 7.37 (dd, J = 8.4, 7.4 Hz, 4H), 7.17-7.14 (m, 2H), 7.12-7.11 (m, 4H), 6.97 (d, J = 8.8 Hz, 2H), 4.59 (q, J = 7.3 Hz, 2H), 1.54 (t, J = 7.3 Hz, 3H). ^{13}C NMR (151 MHz, DMSO) δ 154.34, 149.40, 148.99, 146.22, 144.37, 143.84, 142.10, 134.66, 129.84, 127.63, 125.23, 124.96,

124.42, 124.40, 122.36, 121.35, 116.65, 98.86, 55.41, 16.19. HRMS (MALDI-TOF): m/z: [M+H] + calcd for $C_{32}H_{26}N_3S^+$: 484.1842, found: 484.1846.

Total ROS Detection in Aqueous Solution

A commonly used ROS indicator 2', 7'-dichlorodihydrofluorescein diacetate (DCFH-DA) was utilized to detect the ROS generation of TTCPy-1, TTCPy-2, TTCPy-3 and TTCPy-4 in aqueous solution under white light irradiation (16 mW cm^{-2}). Briefly, 0.5 mL DCFH-DA in ethanol (1 x 10^{-3} M) was added to 2 mL 1 x 10^{-2} M NaOH and allowed to stir at room temperature for 30 min. Then the hydrolysate was neutralized with 10 mL of 1 x PBS at pH 7.4, and kept in dark before use. By the time, DCFH-DA was hydrolyzed to DCFH. Then the ROS indicator (4 x 10^{-5} M) in PBS was further diluted to 5 x 10^{-6} M in the sample solution of TTCPy-1, TTCPy-2, TTCPy-3 and TTCPy-4 (1 x 10^{-6} M) for measurement by PL instrument (Edinburgh FS5 fluorescence spectrophotometer). The fluorescence of 2', 7'-dichlorofluorescein triggered by TTCPy-1-, TTCPy-2-, TTCPy-3- and TTCPy-4 sensitized ROS under white light irradiation were measured at different time intervals. The PL spectra were measured with excitation at 488 nm and emission was collected from 500 to 620 nm. The fluorescence intensity at 525 nm was recorded to indicate the generation rate of total ROS.

$^{\bullet}$OH Detection in Aqueous Solution

The $^{\bullet}OH$ generation potency was evaluated by using Hydroxyphenyl fluorescein (HPF) as an indicator. The stock solution of HPF (5 mM in DMF) was diluted to 5 μM in the sample solution of TTCPy-1, TTCPy-2, TTCPy-3 and TTCPy-4 (1 μM) in PBS buffer. The fluorescence signal of HPF was monitored at different time intervals in a range of 500-620 nm with the excitation wavelength at 490 nm after the solution was irradiated by white light irradiation (16 mW cm^{-2}). The fluorescence intensity at 515 nm was recorded to indicate the generation rate of $^{\bullet}OH$.

$O_2^{-\bullet}$ Detection in Aqueous Solution

The $O_2^{-\bullet}$generation measurements were performed using Dihydrorhodamine 123 (DHR123) as an indicator. The stock solution of DHR 123 (5 mM) was diluted to 5 μM in the sample solution of TTCPy-1, TTCPy-2, TTCPy-3 and TTCPy-4 (1 μM) in PBS. The fluorescence signal of DHR 123 was monitored at different time intervals in a range of 500-620 nm with the excitation wavelength at 495 nm after the solution was irradiated by white light irradiation (16 mW cm^{-2}). The fluorescence intensity at 526 nm was recorded to indicate the generation rate of $O_2^{-\bullet}$.

$^1O_2$ Detection in Aqueous Solution

The $^1O_2$ generation was first assessed by employing Singlet Oxygen Sensor Green (SOSG) as an indicator. The stock solution of SOSG (5 mM) was diluted to 5 μM in the sample solution of TTCPy-1, TTCPy-2, TTCPy-3 and TTCPy-4 (1 μM) and Rose Bengal (RB) (1 μM) in PBS buffer. The fluorescence signal of SOSG was monitored at different time intervals in a range of 500-620 nm with the excitation wavelength at 488 nm after the solution was irradiated by white light irradiation (16 mW cm^{-2}). The fluorescence intensity at 525 nm was recorded to indicate the generation rate of $^1O_2$. For the $^1O_2$ detection indicated by 9,10-anthracenediyl-bis(methylene)-dimalonic acid (ABDA), the stock solution of ABDA (20 mM) was diluted to 20 μM in the sample solution of AIEgens (1 μM) and RB (1 μM) in PBS buffer. The absorption spectra of ABDA were monitored in a range of 330-450 nm after the solution was irradiated by white light irradiation (16 mW cm^{-2}). The absorbance decrease of ABDA at 378 nm was recorded to indicate the decomposition rates of ABDA ($^1O_2$ generation rate).

ESR Analysis

ESR analysis was carried out to confirm the generation of •OH using DMPO as spin-trap agent. The working samples containing 70.8 × 10^{-3} M DMPO in H_2O and 5 × 10^{-4} M of TTCPy-1, TTCPy-2, TTCPy-3 and TTCPy-4 were injected quantitatively into quartz capillaries, and the spectra of spin was monitored before and after the solution was irradiated by white light (100 mW cm^{-2}) for 1 min. ESR analysis was carried out to confirm the

generation of $O_2^{-\bullet}$ using DMPO as spin-trap agent. The working samples containing 70.8 × 10^{-3} M DMPO in MeOH and 5 × 10^{-4} M of TTCPy-1, TTCPy-2, TTCPy-3 and TTCPy-4 were injected quantitatively into quartz capillaries, and the spectra of spin was monitored before and after the solution was irradiated by white light (100 mW cm^{-2}) for 1 min.

Theoretical calculations

The ground-state (S_0) geometries of four AIE-PSs (TTCPy-1, TTCPy-2, TTCPy-3 and TTCPy-4) were all optimized at theoretical level of B3LYP/6-31G (d, p), energies of the first singlet and triplet excited states were then calculated based on the optimized S_0 geometries at the (TD) CAM-B3LYP/6-31G (d, p) level. All the calculations were manipulated by the Gaussian 16 suite.

Cyclic Voltammetry Measurement

Cyclic voltammograms experiment was conducted by using three-electrode system. A platinum-carbon compound electrode was used as working electrode, the Pt wire electrode and the calomel electrode were used as the auxiliary electrode and reference electrode, respectively. The measurement was conducted in dichloromethane containing 0.1 M tetrabutylammonium hexafluorophosphate. The scan range was determined from -0.4 V to 1.6 V and the scan rate was optimized as 50 mV/s. Fc/Fc^+ was used as external reference.

Bacteria Culture

A single colony of bacteria (*E. coli, E. coli* TOP10, MDR *E. coli*, and MRSA) on LB agar was transferred to 2 mL of LB liquid culture medium and grown at 37 ℃ with a shaking speed of 200 rpm overnight. Bacteria were harvested by centrifuging at 8000 rpm for 3 min and washed twice with PBS (pH = 7.4). After removal of the supernatant, the remaining bacteria were resuspended with PBS, and diluted to an optical density of 1.0 at 600 nm (OD_{600} = 1.0 with about 10^9 CFU mL^{-1}).

Bacteria Staining and Imaging

After harvesting bacteria by centrifugation, 5×10^8 CFU mL^{-1} of *E. coli* and MRSA were added with 1 mL PBS containing 10×10^{-6} M TTCPy-1, TTCPy-2, TTCPy-3 and TTCPy-4. After dispersion with vortex, the bacteria were incubated at 37 °C with a shaking speed of 200 rpm for 15 min and 10 min, respectively. To capture fluorescence images, 1 µL of stained bacteria solution was transferred to a piece of glass slide and then covered by a coverslip. The images were collected using a confocal laser scanning microscope (LSM900, Carl Zeiss, Germany). Capture conditions: AIE-PSs: λ_{ex} = 488 nm and λ_{em} = 600–700 nm.

Antimicrobial Assay

For the light-induced toxicity experiment, 10^7 CFU mL^{-1} bacteria (MRSA) were dispersed in the solutions containing TTCPy-1, TTCPy-2, TTCPy-3 and TTCPy-4 (0 x 10^{-6}, 0.25 x 10^{-6}, 0.5 x 10^{-6}, 1 x 10^{-6} M) 10^7 CFU mL^{-1} bacteria (*E. coli*, *E. coli* TOP10, MDR *E. coli*) were dispersed in the solutions containing TTCPy-1, TTCPy-2, TTCPy-3 and TTCPy-4 (0 x 10^{-6}, 2 x 10^{-6}, 5 x 10^{-6}, 10 x 10^{-6} M), and incubated at 30 °C with a shaking speed of 200 rpm for 30 min or under white light irradiation for 10 min (16 mW cm^{-2}). Then, the treated bacteria were washed and diluted in PBS, from which 100 μL (10^4 CFU mL^{-1}) bacteria were sprayed onto a LB agar plate. Later, all the LB agar plates with treated bacteria were cultured at 37 °C for 16-24 h. The images of the plates taken by a digital camera were used for counting the number of colony-forming units (CFU).

FE-SEM Analysis

Bacterial (*E. coli*, MDR *E. coli* and MRSA) cultures at midlog phase in LB medium were collected and resuspended into PBS at density of 5 x 10^8 CFU mL^{-1}. After treatment of *E. coli* (10 × 10^{-6} M), MDR *E. coli* (10 × 10^{-6} M) and MRSA (5 × 10^{-6} M) with TTCPy-1, TTCPy-2, TTCPy-3 and TTCPy-4 with or without light irradiation for 10 min, the bacteria were collected and fixed with 2.5% glutaraldehyde solution overnight. After fixation, the samples were washed with PBS for twice and dehydrated by 30%, 50%, 70%, 80%, 90%, 95%, and 100% (v/v, in water) ethanol in sequence for 15 min each. 2 μL of bacterial suspensions were

added onto clean silicon. The samples were coated with Au before high resolution scanning electron microscope (HR-SEM) (APREO S, thermo scientific, Netherlands) analysis.

TEM Analysis

5×10^{8} CFU mL^{-1} *E. coli* (10×10^{-6} M) and MRSA (5×10^{-6} M) were dispersed in 1 mL of PBS solution in 1.5 mL microcentrifuge tube. The TTCPy-treated *E. coli* (10 µM) and MRSA (5 µM) solution was incubated at 37 °C with a shaking speed of 200 rpm for 30 min and then exposed to white light for 10 min. The TTCPy-treated bacteria were harvested by centrifuging at 8000 rpm for 3 min and fixed by electron microscope fixing solution at 4°C. The TTCPy-treated bacteria samples for TEM characterization were prepared according to the previous reported procedure.[2] The TTCPy-treated bacteria were characterized by TEM (HT7700, Hitachi, Japan).

Cell culturing

HUVECs and NIH-3T3 cells were cultured in Dulbecco's Modified Eagle Medium (DMEM), respectively. All the cells were grown in the media which supplied with 10% fetal bovine serum (FBS), 100 U/mL penicillin and 100 µg/mL streptomycin in a humidified incubator at 37 °C with 5% CO_2 and subcultured every two or three days.

Cell viability via MTT Assay

Cells were grown on a 96-well plate at a density of 1x 10^4 cells per well (200 µL) and incubated for 24 h. After being incubated with TTCPy-1, TTCPy-2, TTCPy-3 and TTCPy-4 at different concentrations (0, 0.3125, 0.625,1.25, 2.5, 5, 10 × 10^{-6} M) for 30 min, the cells were exposed to white light irradiation (16 mW·cm^{-2}) for 10 min, and then incubated for 24 h. 100 µL of fresh DMEM medium containing 10 µL 3-(4,5-Dimethylthiazol-2-yl)-2,5-Diphenyltetrazolium Bromide) (MTT) (5 mg/mL) solution was added to each well after removal of the cell medium, and the cells were incubated for 4 h. 100 µL DMSO was added to each well after the removal of the MTT solution. The absorbance at 570 nm was recorded by a microplate reader (Perkin-Elmer Victor3t) and the cells without treatment served as control.

***In Vivo* Anti-Infection Assay**

Normal 8-10 weeks male Wistar rats with an average weight of 300 g (Beijing Vital River Laboratory Animal Technology Co. Ltd, China) were used in the *in vivo* experiments. All procedures involving animal use were approved by the Animal Ethical and Welfare Committee of Shenzhen University. The Wistar rats were randomly divided into five groups: (1) MRSA bacteria-infected group with no treatment; (2) MRSA bacteria-infected group with TTCPy-3 in darkness; (3) MRSA bacteria-infected group with TTCPy-3 plus white light

irradiation treatment; (4) MDR *E. coli* bacteria-infected group with no treatment; (5) MDR *E. coli* bacteria-infected group with TTCPy-3 plus white light irradiation. The rats were anesthetized by injection of 1% pentobarbital sodium saline solution (5 $mL \cdot kg^{-1}$). Full-thickness skin injures on each rat were prepared by removing a 2.0 cm diameter circular patch of skin on rat. Bacterial suspension (100 μL, 5 x 10^8 $CFU \cdot ml^{-1}$) was dripped on the surface of wounds, and the bacterial suspension were kept in the wounds for 30 minutes. 10 × 10^{-6} M (MDR *E. coli*) or 5 × 10^{-6} M (MRSA) of TTCPy-3 in PBS solution was sprayed on the bacteria-infected wounds for 30 min, and treated with or without white-light irradiation (16 $mW \cdot cm^{-2}$) for 10 min. The rats were separately fed in different cages to facilitate the healing of wound after operation. The wound sizes were imaged by a video camera and calculated at designated time intervals.

Histological Analysis

Histological analysis of the wounds was carried out on day 14 postoperation. The wound tissues were collected and fixed in 4% formaldehyde solution for overnight at 4°C. The pathological sections of wound tissues were analyzed by HE, Masson and CD31 staining. Histological images were taken using sectioning scanner (Pannoramic DESK, P-MIDI, P250; 3D HISTECH, Hungary). The images were analyzed by a pannoramic scanner.

Statistical Analysis

The reported values were expressed as mean ± SD. The Origin 8 software was used for graphic drawing. The statistical significance of differences between groups was performed by using pair-sample t-testing method on the Origin 8.0 software. A value of $P < 0.05$ was considered significant and were indicated with asterisks: $*P < 0.05$ and $**P < 0.001$. Each experiment included at least three repetitions.

Figures and tables

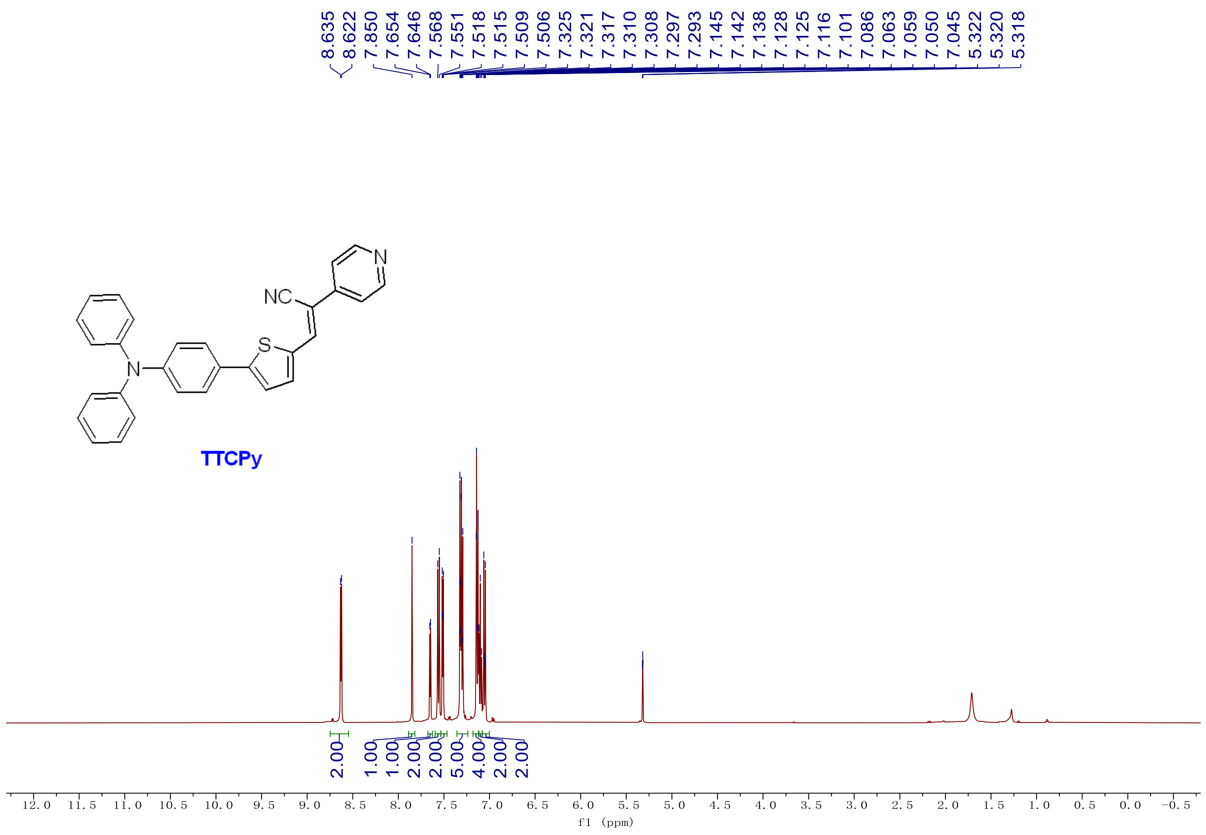

Figure S1. ^{1}H NMR spectrum of TTCPy.

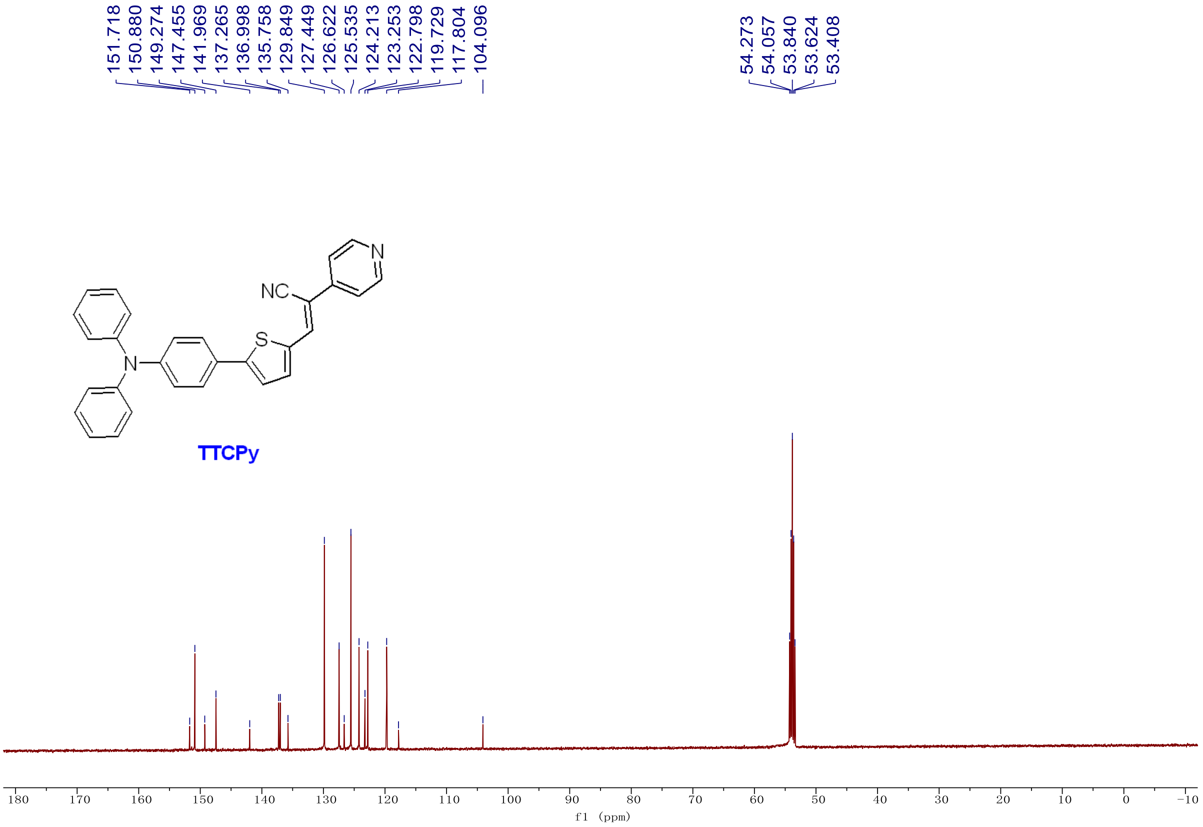

Figure S2. ^{13}C NMR spectrum of TTCPy.

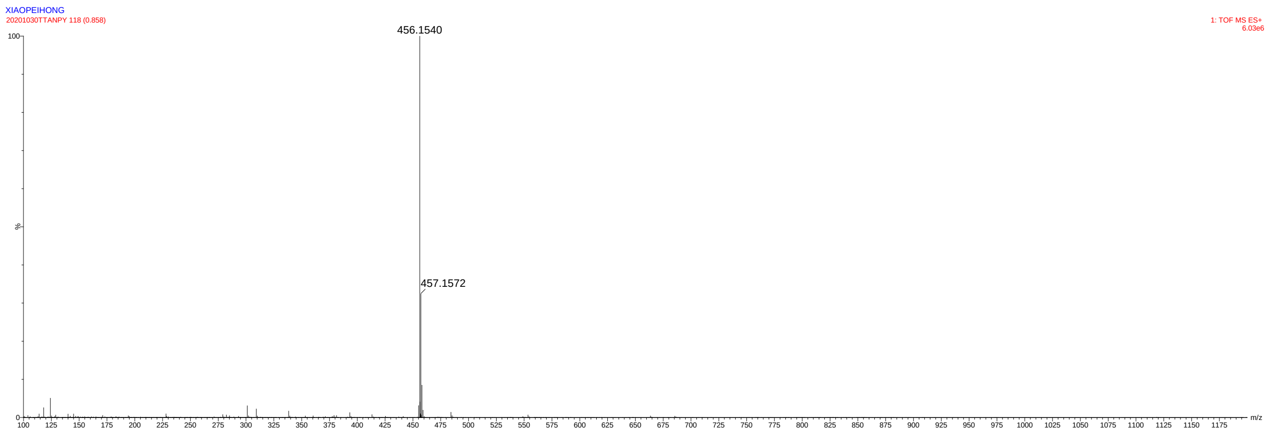

Figure S3. HRMS spectrum of TTCPy.

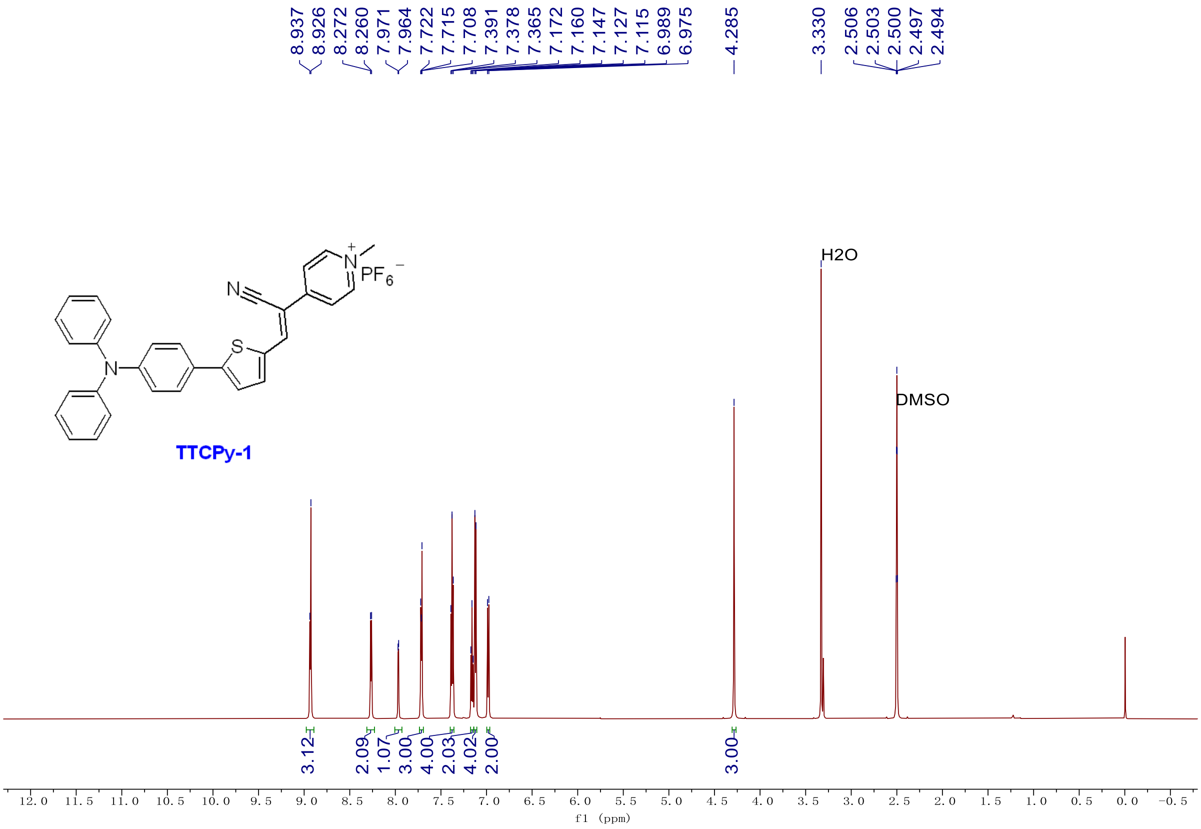

Figure S4. ^{1}H NMR spectrum of TTCPy-1.

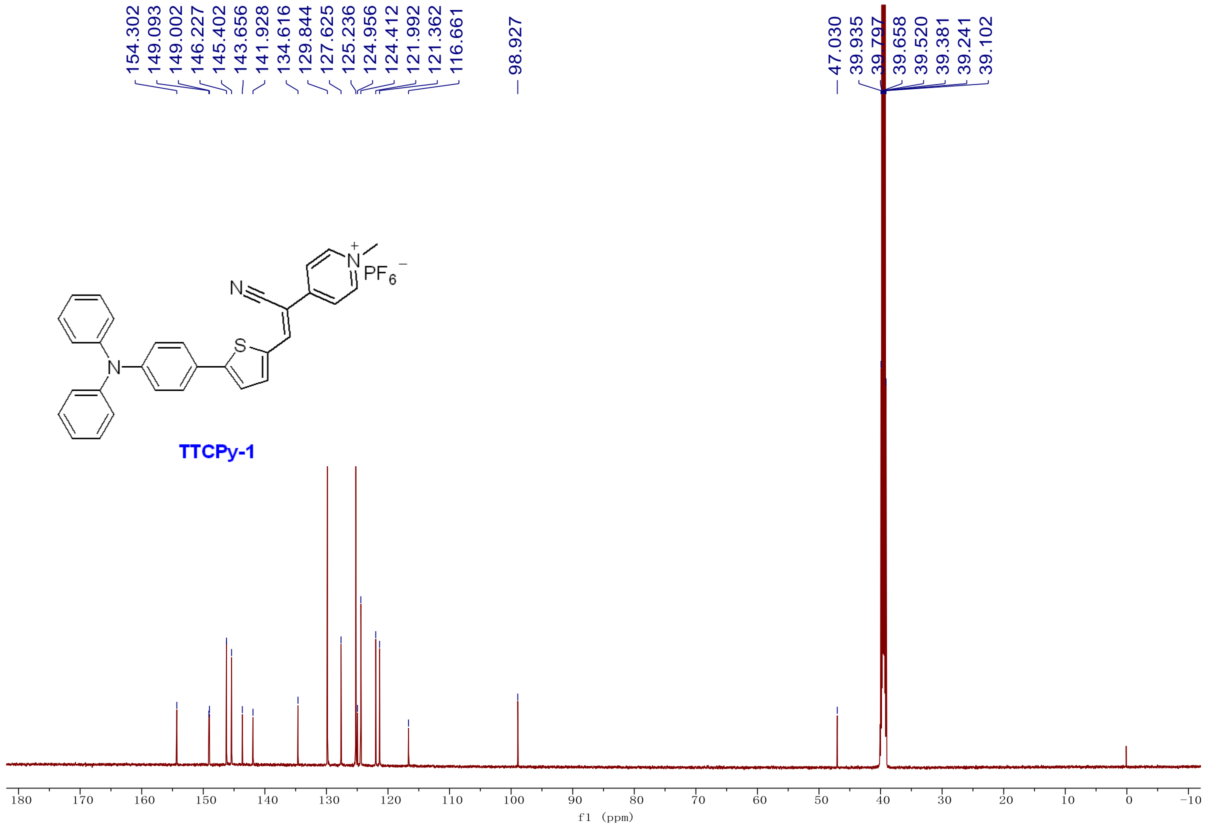

Figure S5. ^{13}C NMR spectrum of TTCPy-1.

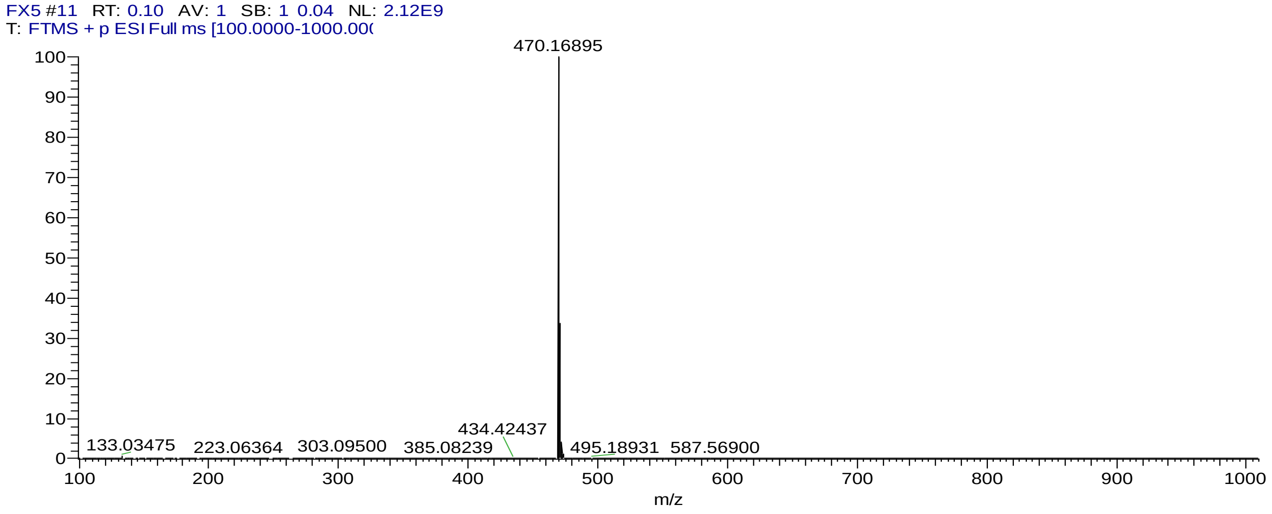

Figure S6. HRMS spectrum of TTCPy-1.

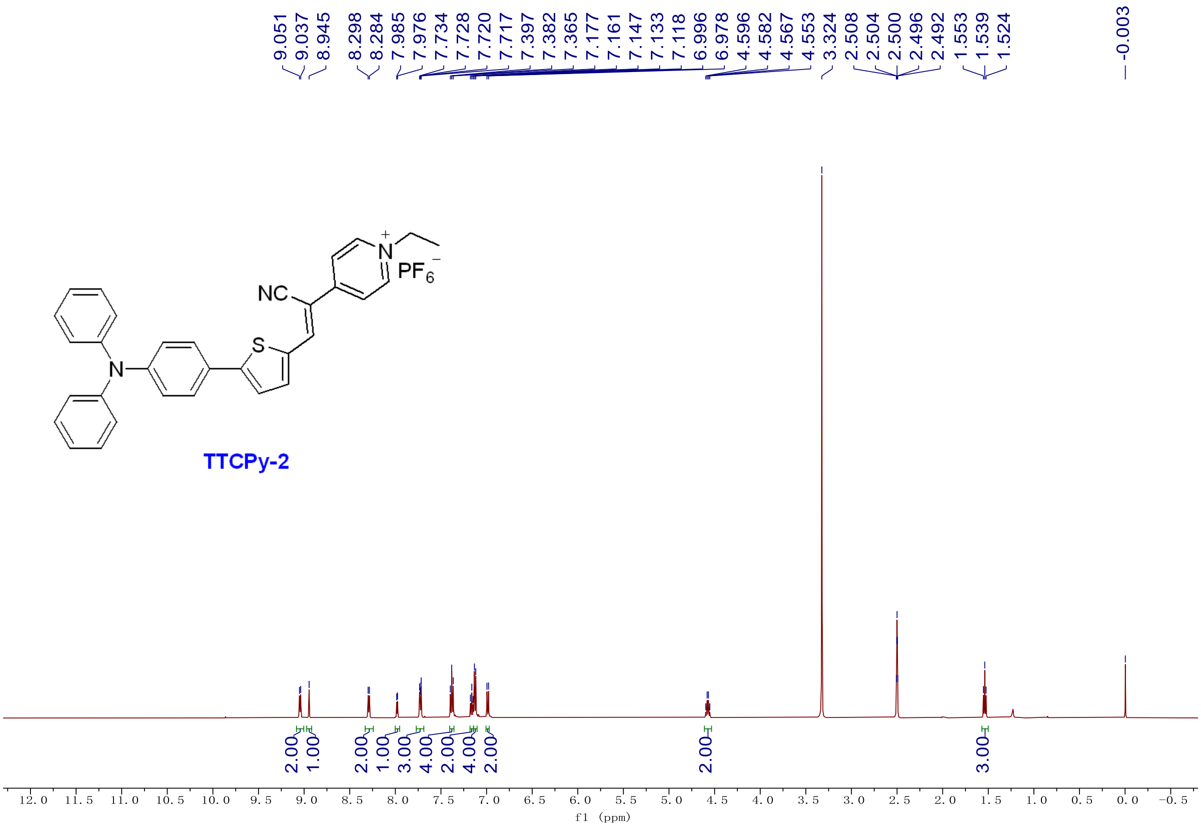

Figure S7. ^{1}H NMR spectrum of TTCPy-2.

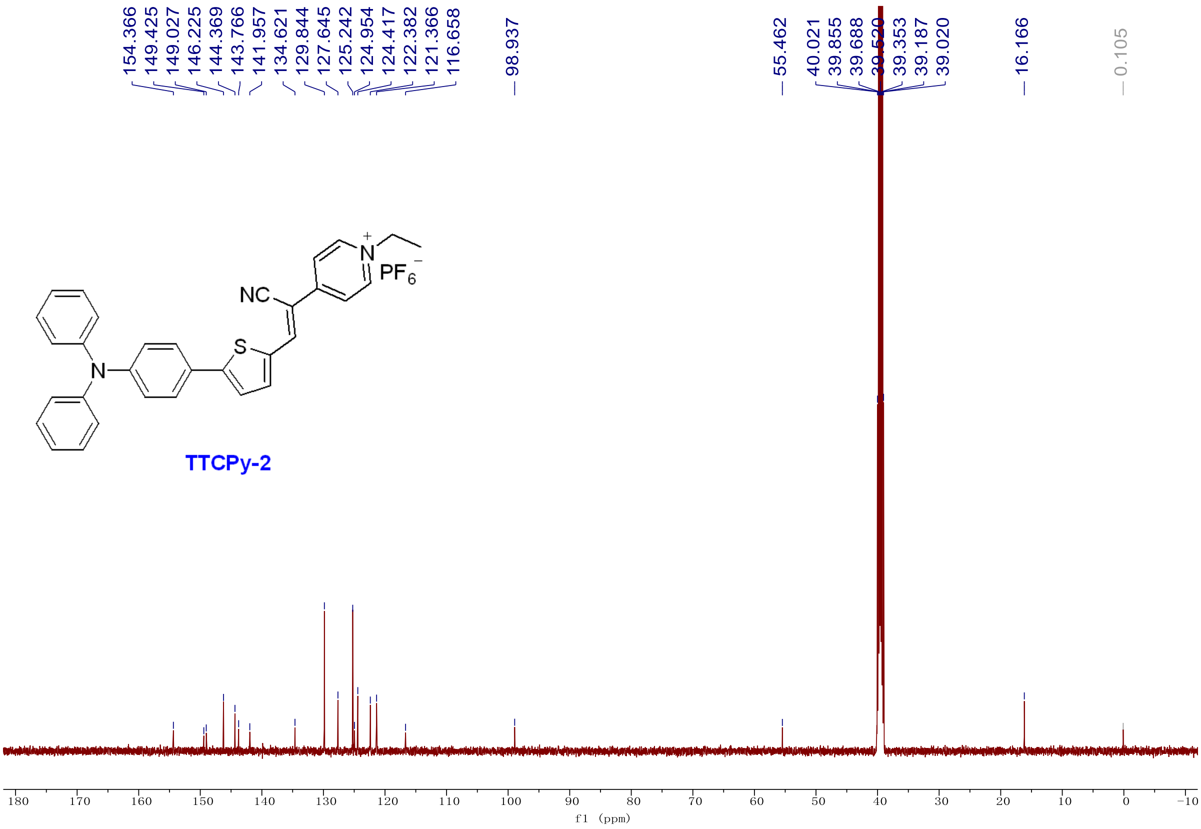

Figure S8. ^{13}C NMR spectrum of TTCPy-2

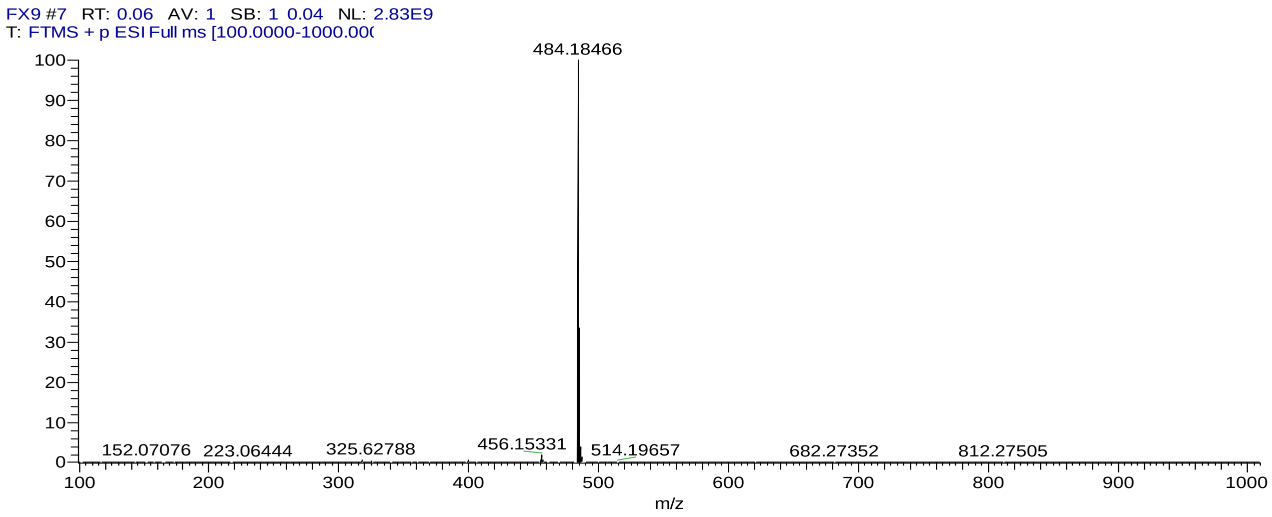

Figure S9. HRMS spectrum of TTCPy-2.

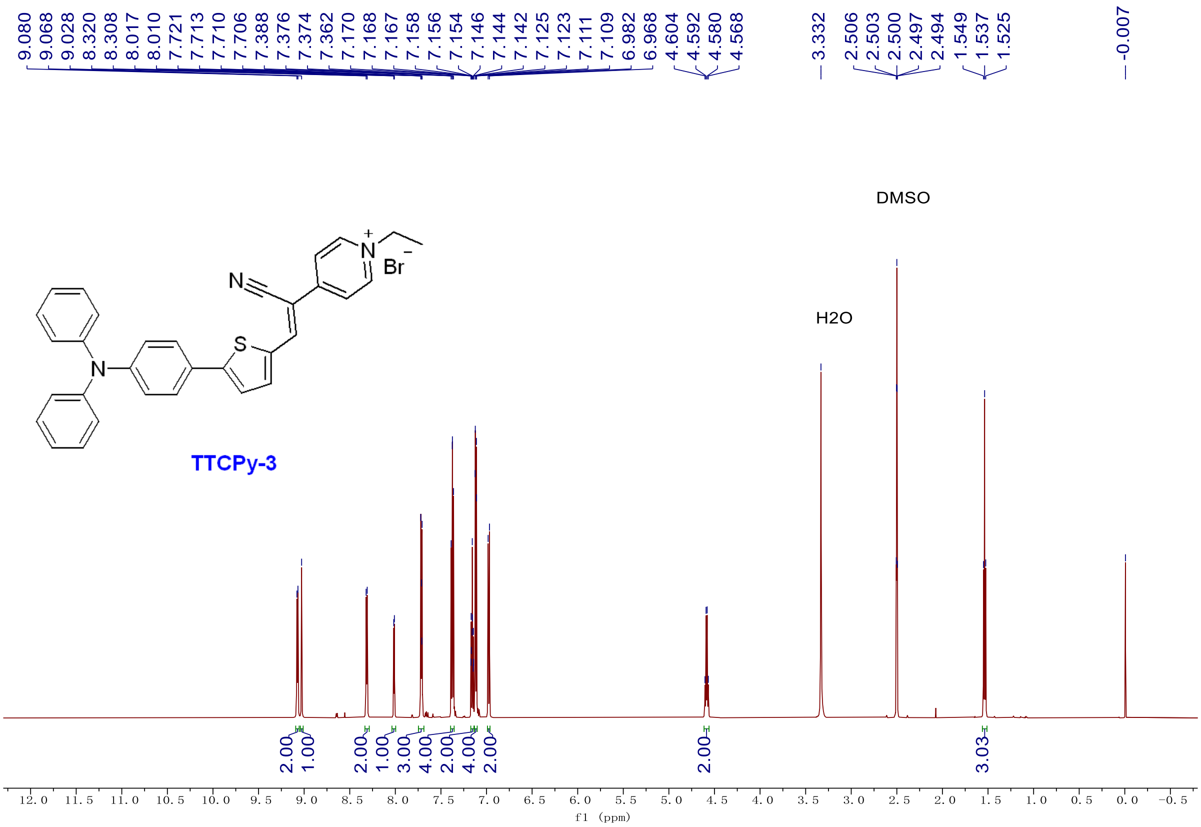

Figure S10. ^{1}H NMR spectrum of TTCPy-3.

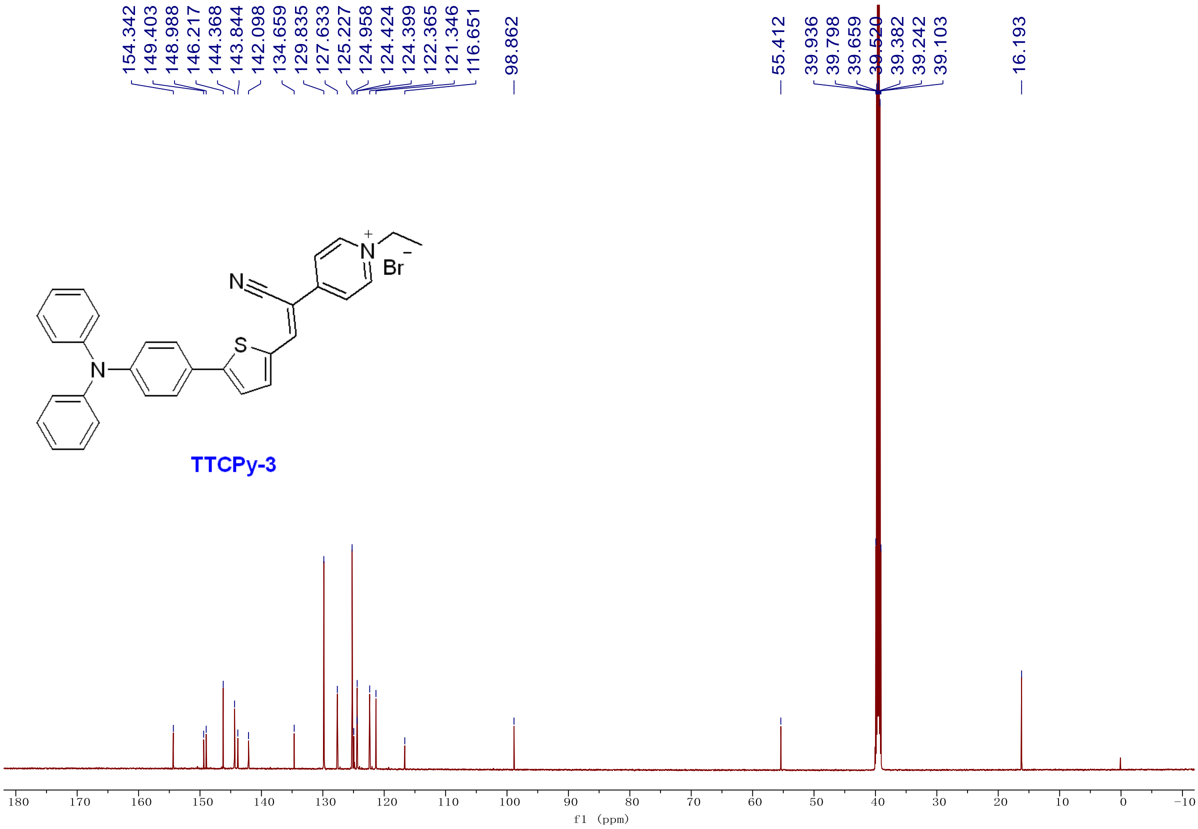

Figure S11. ^{13}C NMR spectrum of TTCPy-3.

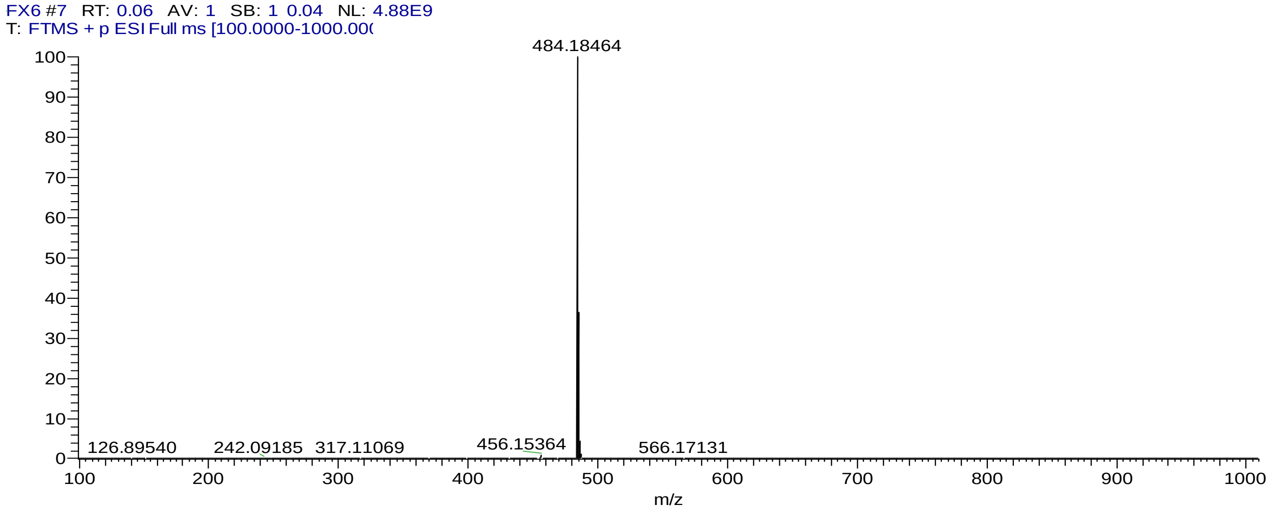

Figure S12. HRMS spectrum of TTCPy-3.

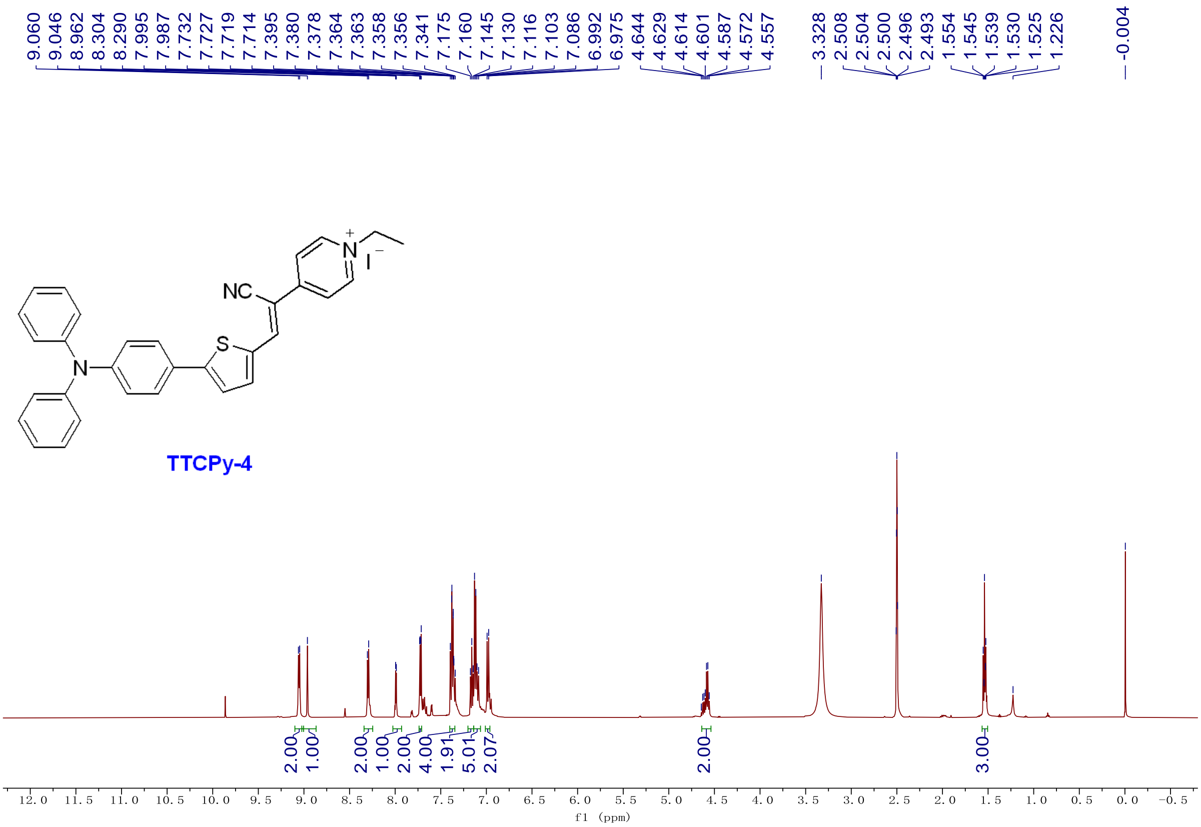

Figure S13. ^{1}H NMR spectrum of TTCPy-4.

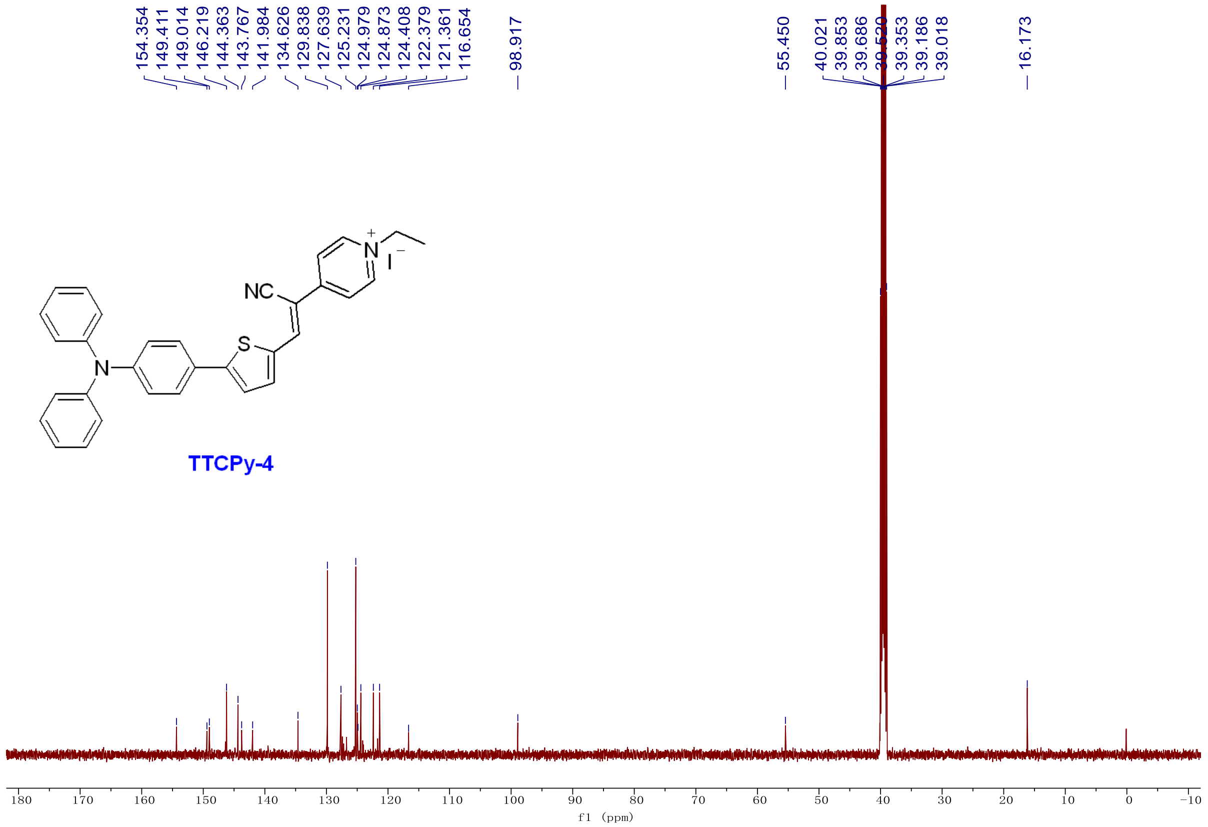

Figure S14. ^{13}C NMR spectrum of TTCPy-4.

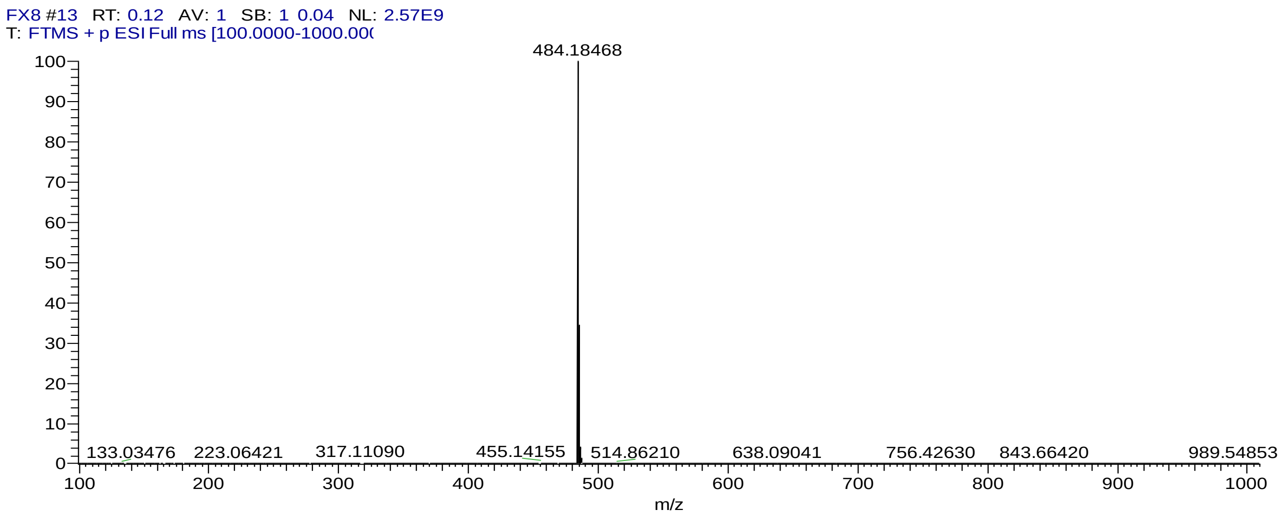

Figure S15. HRMS spectrum of TTCPy-4.

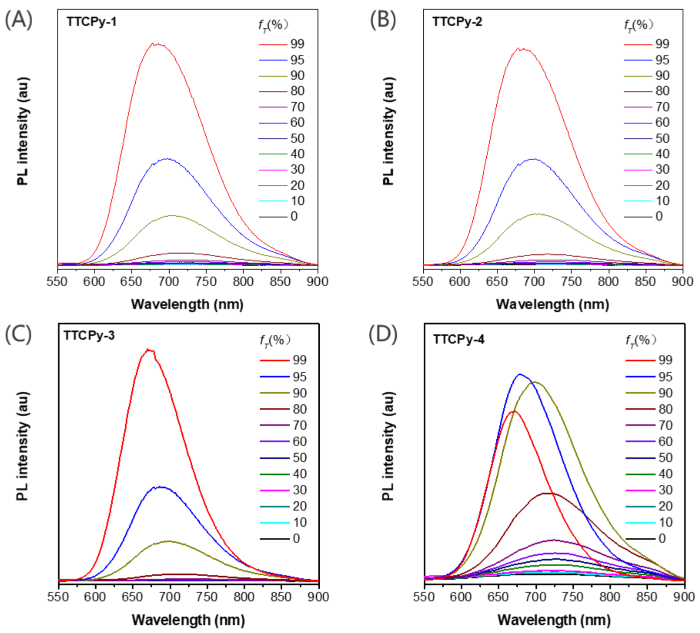

Figure S16. PL spectra of (A) TTCPy-1 (10 x 10^{-6} M), (B) TTCPy-2 (10 x 10^{-6} M), (C) TTCPy-4 (10 x 10^{-6} M) in DMSO/Toluene mixtures with different toluene fractions (f_w).

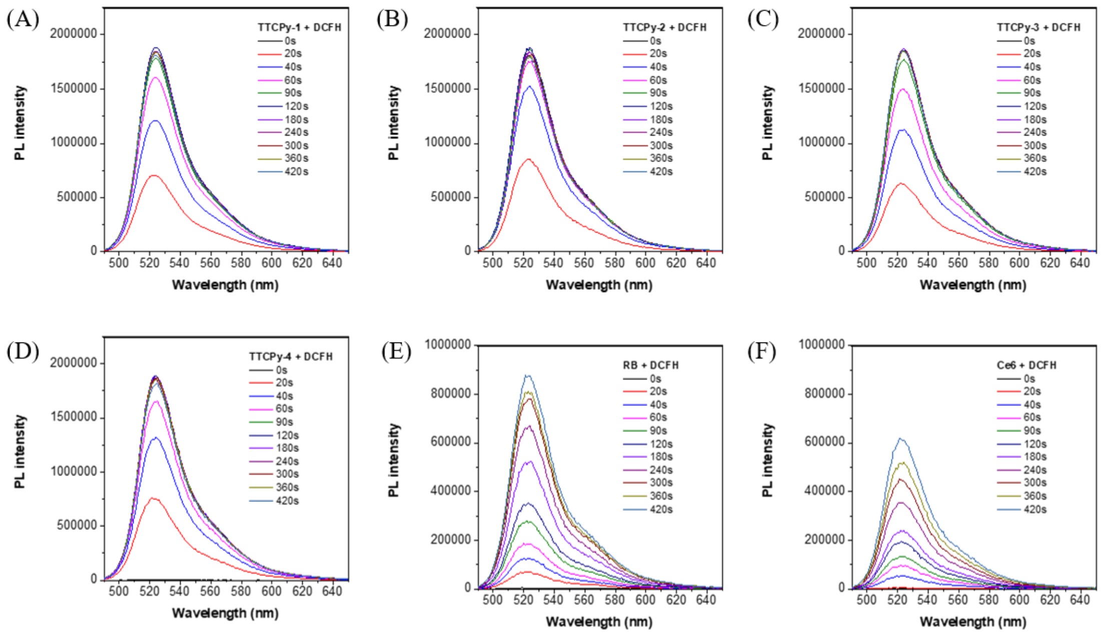

Figure S17. PL spectra of DCFH in PBS in present of (A) TTCPy-1, (B) TTCPy-2, (C) TTCPy-3, (D) TTCPy-4, (E) RB and (F) Ce6 after exposure to white light irradiation with different time. [AIE-PSs or Rose Bengal or Ce6] = 1×10^{-6} M, Light power: 16 mW cm^{-2}.

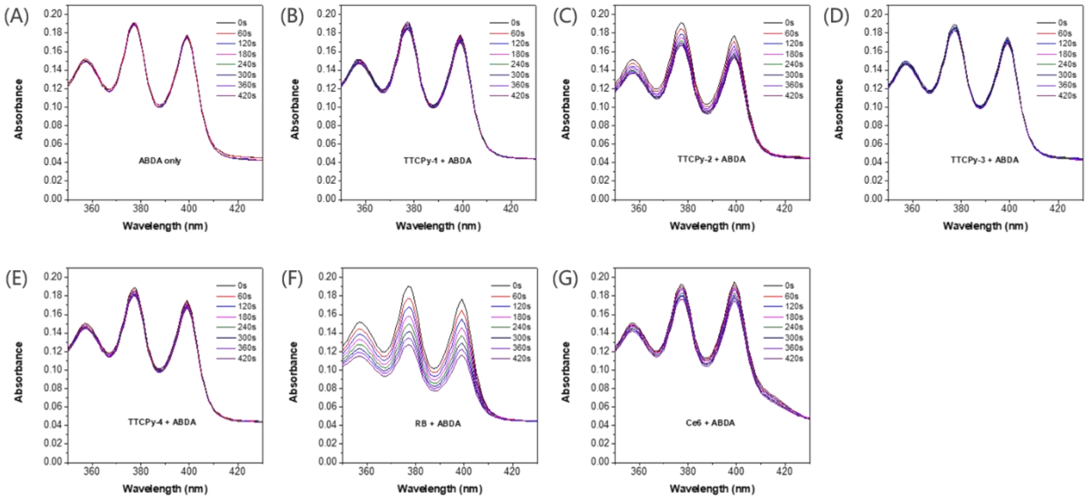

Figure S18. (A) UV-vis spectra of ABDA in the absence of AIE-PSs under white light irradiation in DMSO/water (v:v) = 1/100. UV-vis spectra of ABDA in the presence of (B) TTCPy-1 or (C) TTCPy=2 or (D) TTCPy-3 or (E) TTCPy-4 or (F) Rose Bengal or (G) Ce6 under white light irradiation in DMSO/water (v:v) = 1/100. [AIE-PSs or Rose Bengal or Ce6] = 1×10^{-6} M, [ABDA] = 5×10^{-5} M, time interval for recording the UV-vis spectra: 60 s.

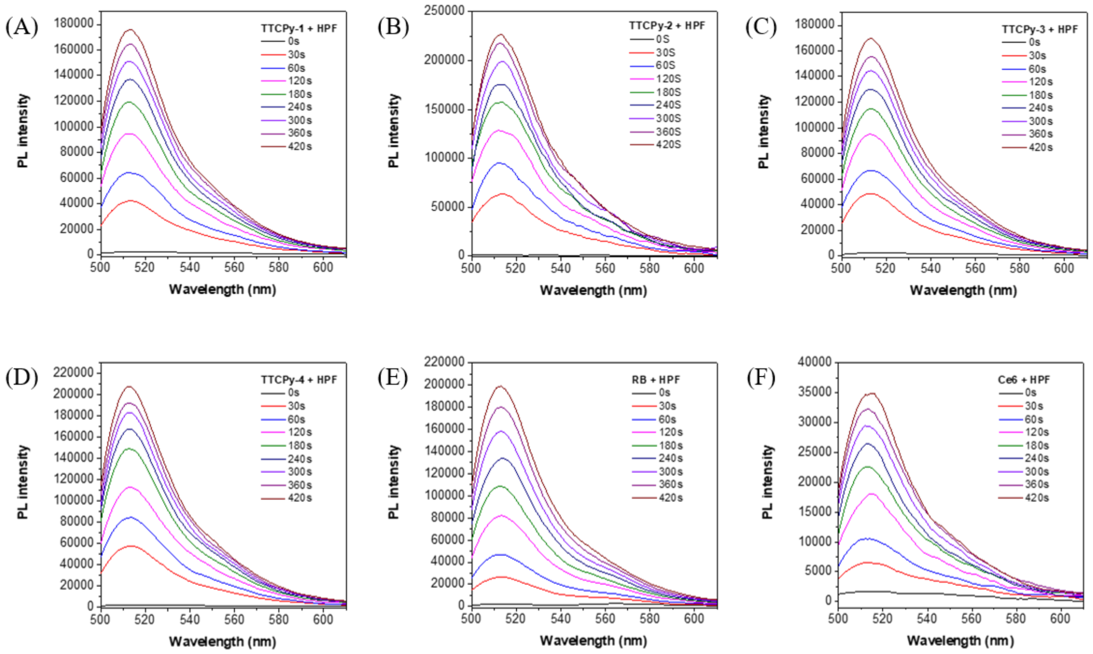

Figure S19. PL spectra of HPF in PBS in present of (A) TTCPy-1, (B) TTCPy-2, (C) TTCPy-3, (D) TTCPy-4, (E) RB and (F) Ce6 after exposure to white light irradiation with different time. [AIE-PSs or Rose Bengal or Ce6] = 1×10^{-6} M, [HPF] = 5×10^{-6} M, Light power: 16 mW cm^{-2}.

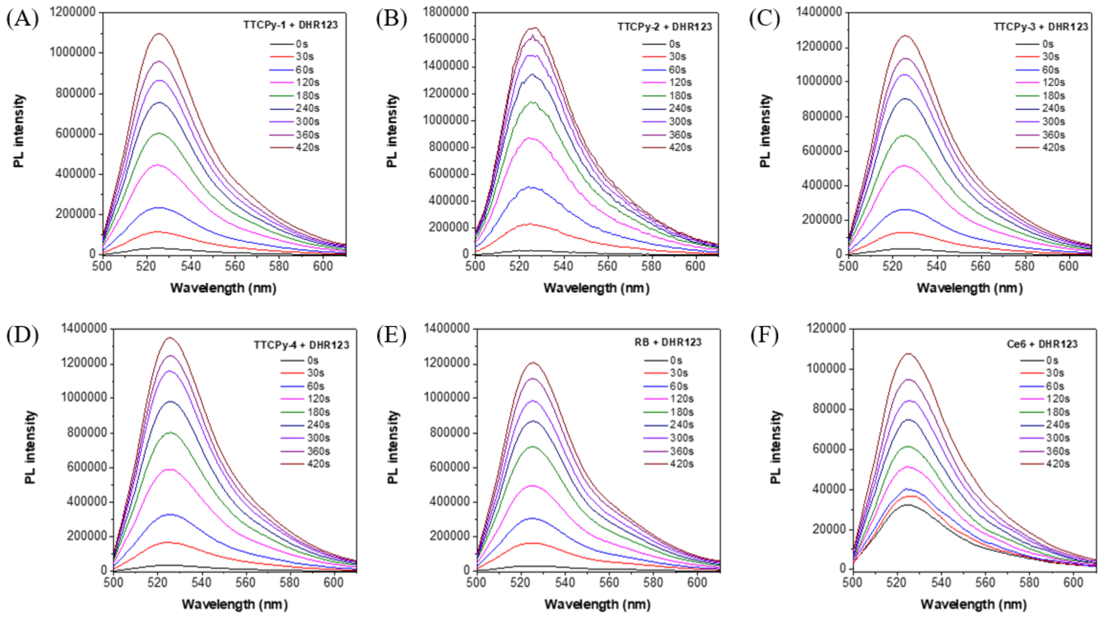

Figure S20. PL spectra of DHR 123 in PBS in present of (A) TTCPy-1, (B) TTCPy-2, (C) TTCPy-3, (D) TTCPy-4, (E) RB and (F) Ce6 after exposure to white light irradiation with

different time. [AIE-PSs or Rose Bengal or Ce6] = 1×10^{-6} M, [DHR 123] = 5×10^{-6} M, Light power: 16 mW cm^{-2}.

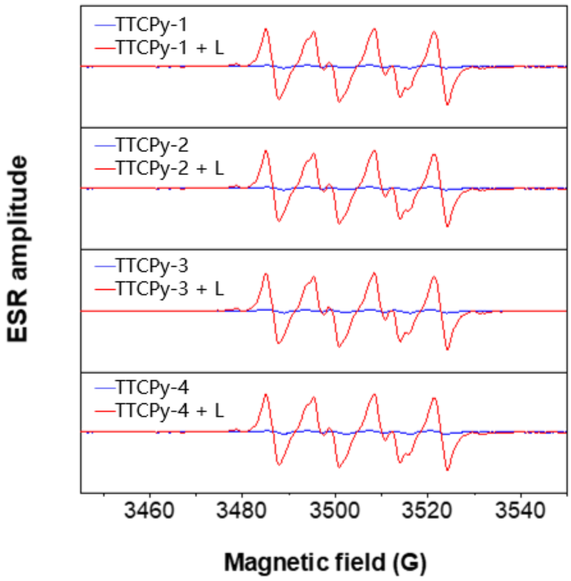

Figure S21. ESR signals of DMPO for Type-I ROS $O_2^{-\bullet}$ characterization in the presence of TTCPy-1, TTCPy-2, TTCPy-3 or TTCPy-4 (0.5 Mm in MeOH) before and after white light irradiation (100 mW cm^{-2}).

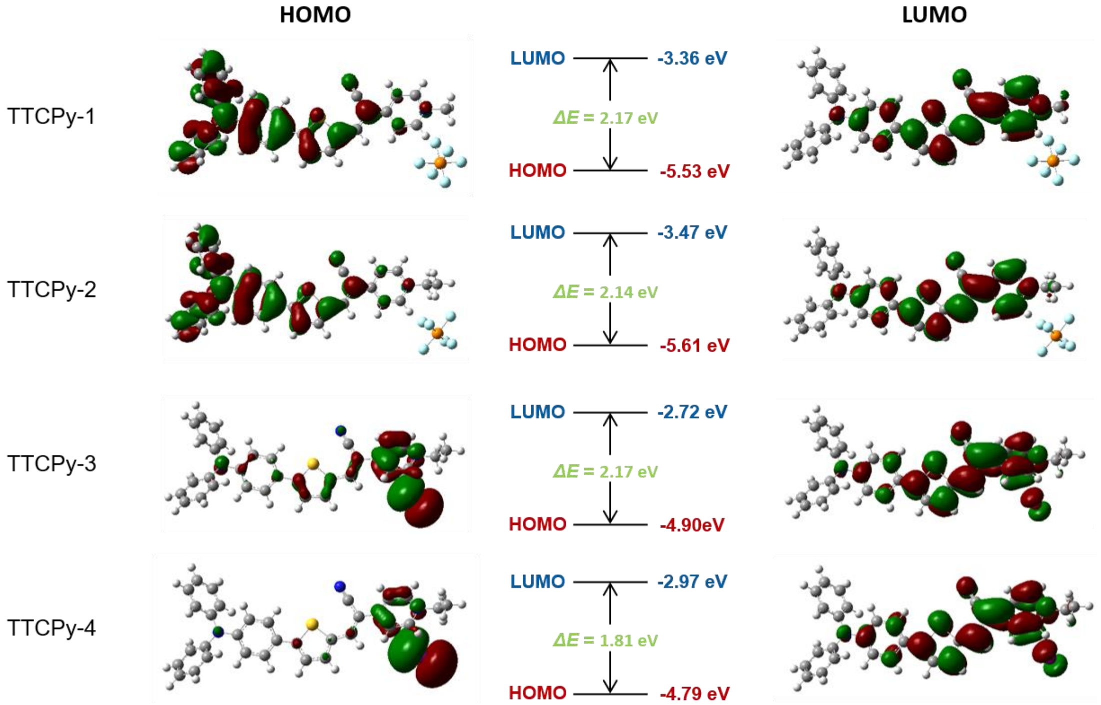

Figure S22. Frontier molecular orbitals for TTCPy-1, TTCPy-2, TTCPy-3 and TTCPy-4. Calculations were performed by density functional theory calculations at the B3LYP-D3/6-31G (d, p) level using the Gaussian 09 program.

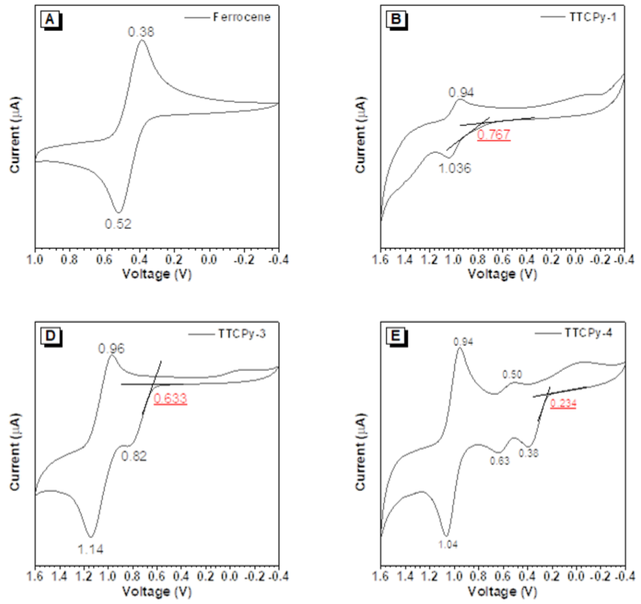

Figure S23. Cyclic voltammograms (CVs) of TTCPy-1, TTCPy-2, TTCPy-3 and TTCPy-4 (1 mM) in CH_2Cl_2 solution. The CVs were determined by using 0.1 M *n*-Bu4NPF6 as the

supporting electrolyte, saturated calomel electrode (SCE) as the reference electrode, Pt disk and Pt wire as counter electrodes, and ferrocene was used as external standard. The scan rate was 50 mV s^{-1}.

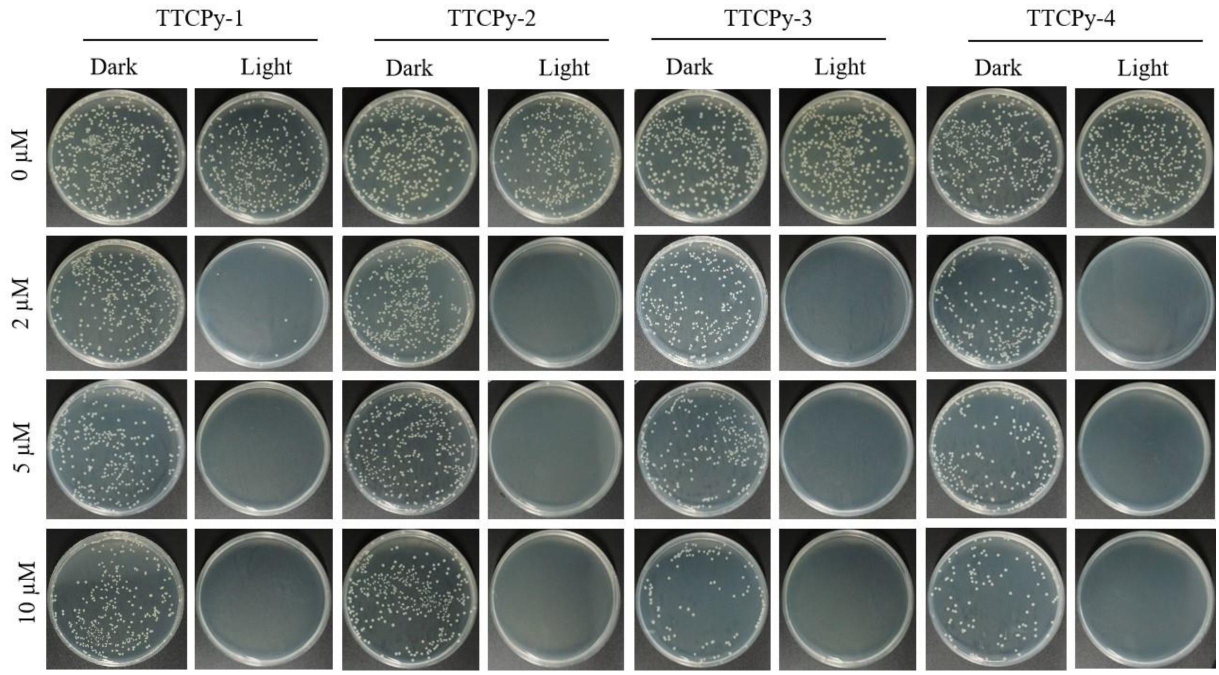

Figure S24. Photodynamic antibacterial activity of TTCPy-1, TTCPy-2, TTCPy-3 and TTCPy-4 on *E. coli*. Photographs of the agar plates of *E. coli* with or without white light (16 mW cm^{-2}).

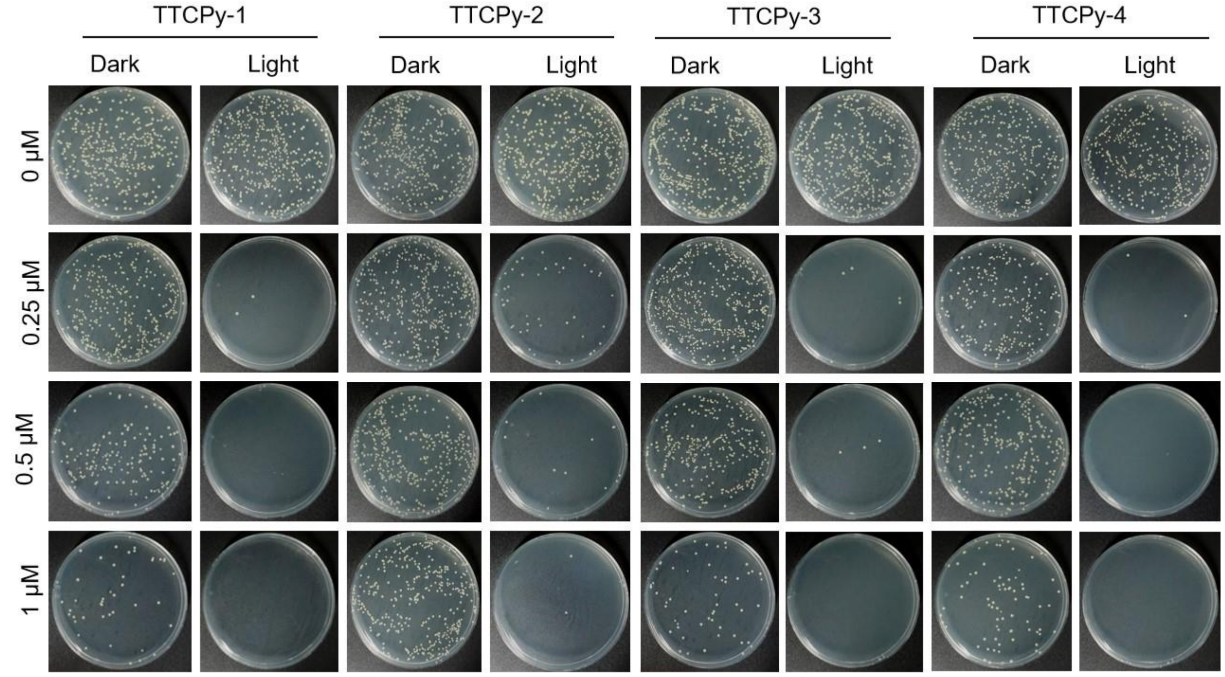

Figure S25. Photodynamic antibacterial activity of TTCPy-1, TTCPy-2, TTCPy-3 and TTCPy-4 on MRSA. Photographs of the agar plates of MRSA with or without white light irradiation (16 mW cm^{-2}).

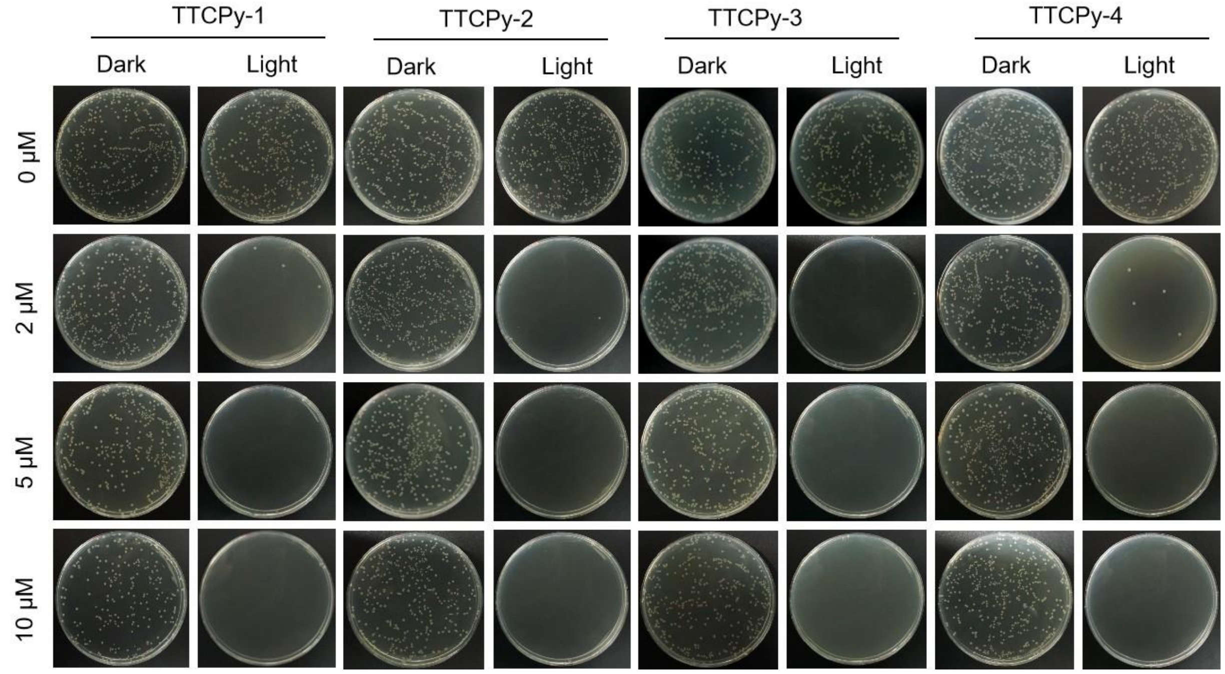

Figure S26. Photodynamic antibacterial activity of TTCPy-1, TTCPy-2, TTCPy-3 and TTCPy-4 on *E. coli* Top10. Photographs of the agar plates of *E. coli* Top10 with or without white light irradiation (16 mW cm^{-2}).

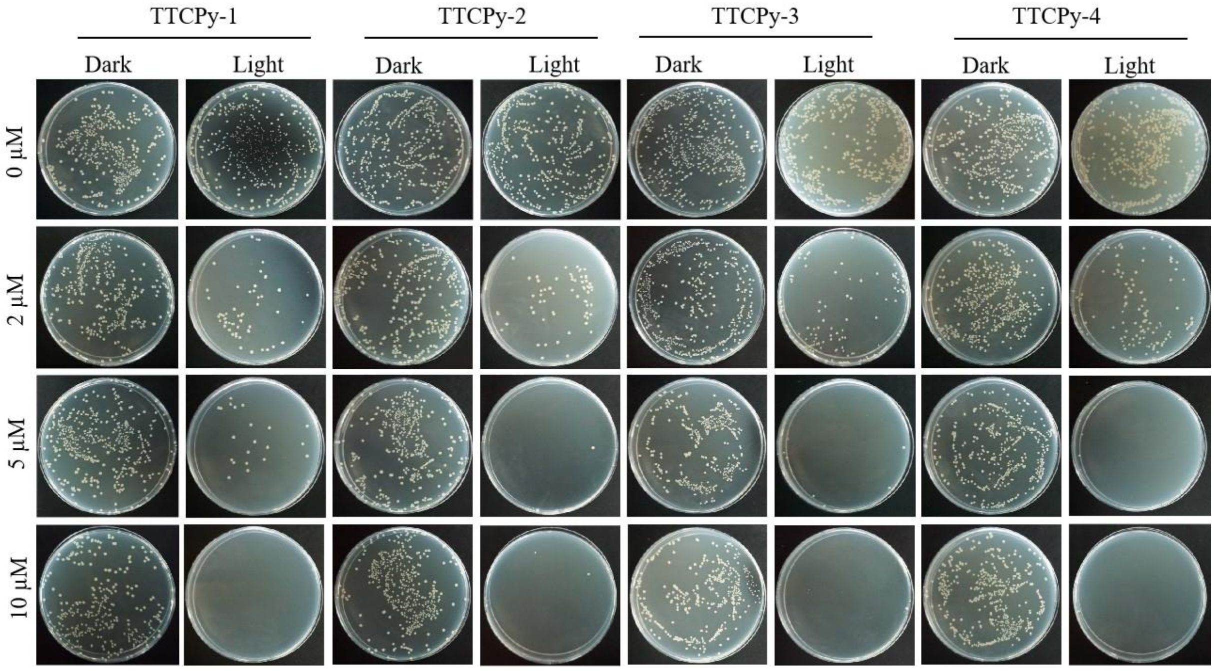

Figure S27. Photodynamic antibacterial activity of TTCPy-1, TTCPy-2, TTCPy-3 and TTCPy-4 on MDR *E. coli*. Photographs of the agar plates of MDR *E. coli* with or without white light irradiation (16 mW cm^{-2}).

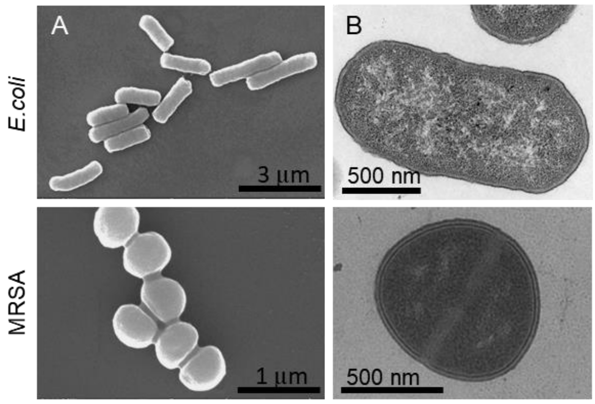

Figure S28. Visualizing light-induced morphological changes of *E. coli* and MRSA upon white light irradiation (16 mW cm^{-2}) by FE-SEM (A) and TEM (B).

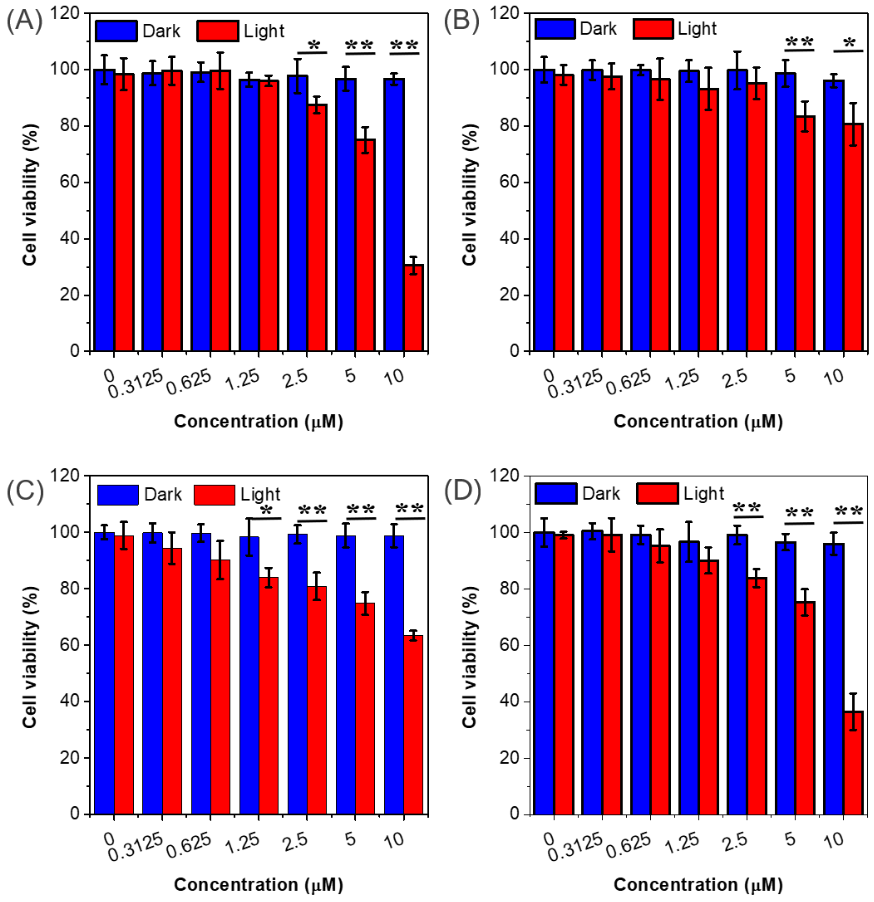

Figure S29. MTT assay of HUVECs treated by different concentrations of (A) TTCPy-1, (B) TTCPy-2, (C)TTCPy-3, and (D)TTCPy-4 in darkness or upon white light irradiation (16 mW cm^{-2}) for 10 min (n = 5). The data show significant statistical differences between cell-treated by TTCPy-1, TTCPy-2, TTCPy-3 and TTCPy-4 with or without light irradiation (16 mW cm^{-2}) respectively ($*P < 0.05$, $**P < 0.001$).

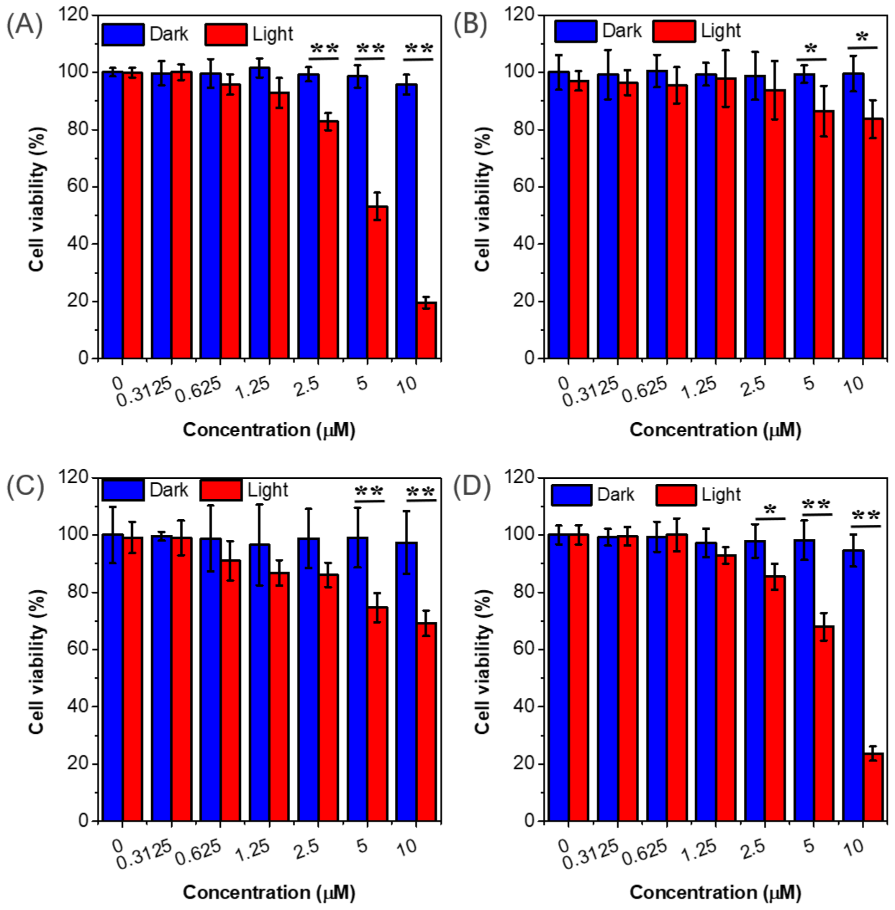

Figure S30. MTT assay of NIH-3T3 treated by different concentrations of (A) TTCPy-1, (B) TTCPy-2, (C)TTCPy-3, and (D)TTCPy-4 in darkness or upon white light irradiation (16 mW cm^{-2}) for 10 min (n = 5). The data show significant statistical differences between cell-treated by TTCPy-1, TTCPy-2, TTCPy-3 and TTCPy-4 with or without light irradiation (16 mW cm^{-2}) respectively (*P < 0.05, **P < 0.001).

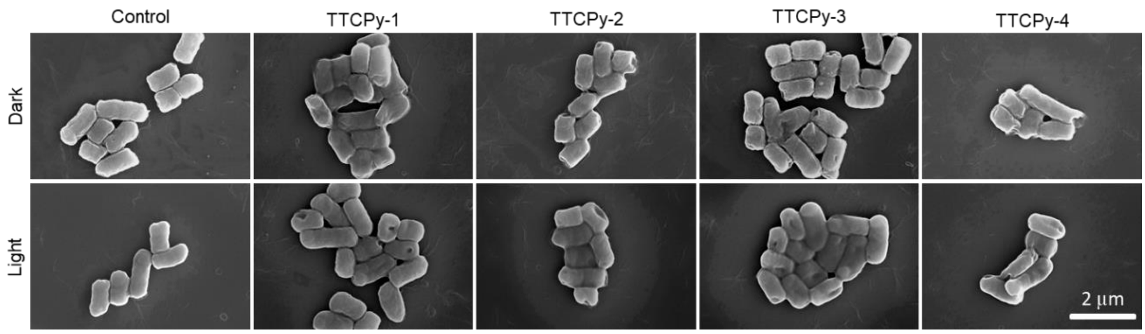

Figure S31. Visualizing TTCPy-1, TTCPy-2, TTCPy-3 and TTCPy-4-induced morphological changes of MDR *E. coli* in darkness or upon white light irradiation (16 mW cm^{-2}) by FE-SEM. Bacteria without treatment were set as control.

Main Text Paragraph.

Table S1. Optical properties of TTCPy-1, TTCPy-2, TTCPy-3 and TTCPy-4.

AIEgens	λ_{abs}[a] (nm)	λ_{em} (nm) Soln (Φ_F)[b,c]	Aggr	Solid (Φ_F)[c,d]	α_{AIE} ($I_{aggr, max}/I_{soln}$)	ΔE_{H-L} [eV][e]	ΔE_{st} [eV][f]
TTCPy-1	532	716 (0.9%)	694 (12.9%)	654 (1.9%)	99.7	2.17	1.22
TTCPy-2	532	677 (0.8%)	701 (12.1%)	650 (1.1%)	83.2	2.14	1.20
TTCPy-3	534	685 (0.5%)	686 (19.2%)	748 (1.1%)	183.9	2.17	1.15
TTCPy-4	534	708 (0.7%)	679 (6.1%)	742 (1.2%)	28.2	1.81	0.74

[a] Absorption maximum in DMSO solutions. [b] Emission maximum in DMSO (10 µM). [c] Fluorescence quantum yield determined by a calibrated integrating sphere. [d] Emission maximum in solid state. [e] The value of HOMO-LUMO energy gap of AIE-PSs. (f)The value of singlet-triplet energy gap of AIE-PSs.

References

[1] G. Niu, X. Zheng, Z. Zhao, H. Zhang, J. Wang, X. He, Y. Chen, X. Shi, C. Ma, R. T. K. Kwok, J. W. Y. Lam, H. H. Y. Sung, I. D. Williams, K. S. Wong, P. Wang and B. Z. Tang, *J. Am. Chem. Soc.* **2019**, *141*, 15111.

[2] Y. Zhao, Y. Tian, Y. Cui, W. Liu, W. Ma, X. Jiang, *J. Am. Chem. Soc.* **2010**, *132*, 12349.
